# Total Synthesis of Septocylindrin B and C-Terminus Modified Analogues

**DOI:** 10.1371/journal.pone.0051708

**Published:** 2012-12-20

**Authors:** Jo Nelissen, Koen Nuyts, Marta De Zotti, Rob Lavigne, Chris Lamberigts, Wim M. De Borggraeve

**Affiliations:** 1 Molecular Design and Synthesis, Department of Chemistry, University of Leuven, Heverlee, Belgium; 2 Department of Chemistry, University of Padova, Padova, Italy; 3 Laboratory of Gene Technology, Biosystems Department, University of Leuven, Heverlee, Belgium; Bioinformatics Institute, Singapore

## Abstract

The total synthesis is reported of the peptaibol Septocylindrin B which is related to the well documented channel forming peptaibol antibiotic Alamethicin. Several analogues were synthesized with a modified *C*-terminus, to investigate the SAR of the terminal residue Phaol. All these peptides were tested for their membrane perturbation properties by fluorescent dye leakage assay and for their antibacterial activity.

## Introduction

**Figure 1:**
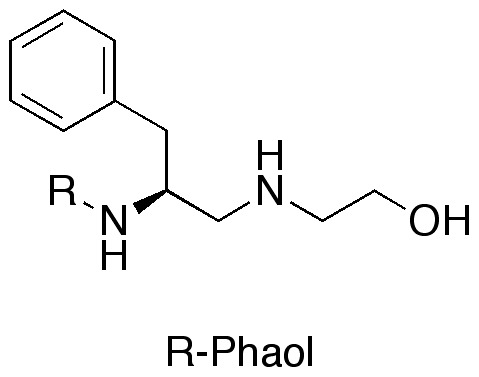
Phaol [2-{[(2*S*)-2-amino-3-phenylpropyl]amino}ethanol]. R-Phaol, R = Ac-UPUAUAQUVUGLUPVUUQQ for Septocylindrin B.

**Figure 2:**
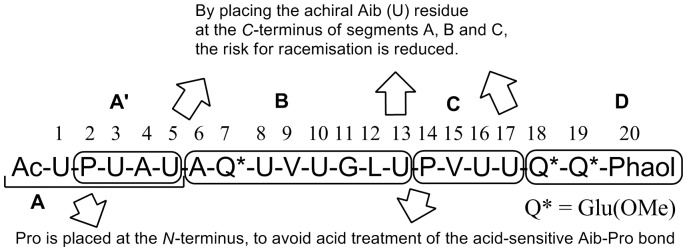
Synthetic approach for Septocylindrin B. A' = residue 2–5, A = residue 1–5, B = 6–13, C = residue 14–17, D = residue 18–20.

In 2007 two new, 20 amino acid long, Phaol-terminated peptaibols, Septocylindrin A and Septocylindrin B were isolated from Septocylindrum *sp.* by Summers *et al* (Figure 1 and 2) [1]. They exhibit significant antibacterial and antifungal activity and are closely related to Alamethicin, one of the most extensively studied peptaibols. Peptaibols are a large class of natural peptides with three characteristic features: a high α-aminoisobutyric acid (Aib) content, an acylated *N*-terminus and a 1,2-amino alcohol at the *C*-terminus [2]. They are known for their membrane-modifying and pore-forming abilities, thereby exhibiting antibacterial and antifungal activity [3]. They promote voltage-dependent ion channel formation in lipid bilayers. The mechanism of this action is yet to be fully understood. Previous studies suggest that simple physico-chemical properties of membrane-active peptides, like aggregation and water/membrane partition, probably play a fundamental role in determining the overall activity [4]. Development of bacterial resistance against this kind of peptides is much less likely in view of their mechanism of action. As a consequence, these peptides are attractive candidates for clinical development.

Phaol (Figure 1) is the amino alcohol present at the *C*-terminus of a number of peptaibols, like Aibellin [5] and Septocylindrin [1]. In view of a total synthesis of these peptides, orthogonally protected Phaol is needed in large amounts. In 2011, Nelissen *et al.* [6] presented a procedure for the large scale synthesis of orthogonally protected Phaol and analogues thereof. The goal of the present work is to exploit these newly synthesized amino alcohol moieties to accomplish the total synthesis of Septocylindrin B and analogues *via* a segment condensation approach. These compounds will then be used to determine the structure-activity relationship (SAR).

## Results and Discussion

### Synthesis of Septocylindrin B

Septocylindrin B is synthesized *via* a segment condensation approach, which is depicted in Figure 2. Septocylindrin B is synthesized by converting the Glu(OMe) residues to Gln residues by aminolysis. The presence of sterically hindered α,α-dialkyl amino acid residues (Aib, U), renders this peptide, like all other peptaibols, less attractive for normal solid phase peptide synthesis. Peggion *et al* [7] published a flexible segment condensation approach for the synthesis of ALM F50/5, one of the components of the complex, natural Alamethicin mixture. They split the peptide into four segments, taking into account the acid sensitive Aib-Pro bond [8], the high tendency of the H-Aib-Pro- dipeptide sequence to cyclize to the corresponding diketopiperazine and the epimerization risk involved in a segment condensation. In particular, to avoid the latter event, each segment was planned so as to have a non-racemizable, achiral Aib residue at the *C*-terminus. A similar approach was used to synthesize Septocylindrin B. We further optimize this approach by splitting the *C*-terminal segment into two parts: **C** and **D**. In this way, the synthesis of Septocylindrin analogues with a modified *C*-terminus is more time efficient, as only the appropriate segment **D** has to be synthesized, while **ABC**-O*t*Bu can be prepared only once on a large scale.

All segments were synthesized using HOAt and EDC as coupling reagents and NMM as a base. Cbz was used for the protection of all *N*-termini and *t*Bu was used for the protection of all *C*-termini. The Cbz group was cleaved off by catalytic hydrogenation, while the *t*Bu group was removed using TFA with the exception of the peptide segments containing the acid sensitive Aib-Pro bond. In this case, the Lewis acid ZnBr_2_ was used [9]. The secondary amine and the alcohol of the Phaol residue were both protected with a Boc group, as described by Nelissen *et al* [6], and deprotected using BiCl_3_ [10]. The glutamic acid residues were used as γ-methyl esters (residue Q* in Figure 2) and subsequently converted to glutamine by treatment with NH_3_.

The single coupling steps in the synthesis of segment **A'** resulted in good yields: about 85% each. Also the synthesis of segment **B** did not pose problems. The coupling reactions proceeded with yields between 76% and 88%. Similarly, the **C** segment was synthesized with yields between 68% to 88% per coupling step. The synthesis of the **D** segment also proceeded smoothly: 91% yield for the synthesis of Cbz-Q*-Phaol(Boc)_2_ and 79% for that of Cbz-Q*Q*-Phaol(Boc)_2_.

**Figure 3:**
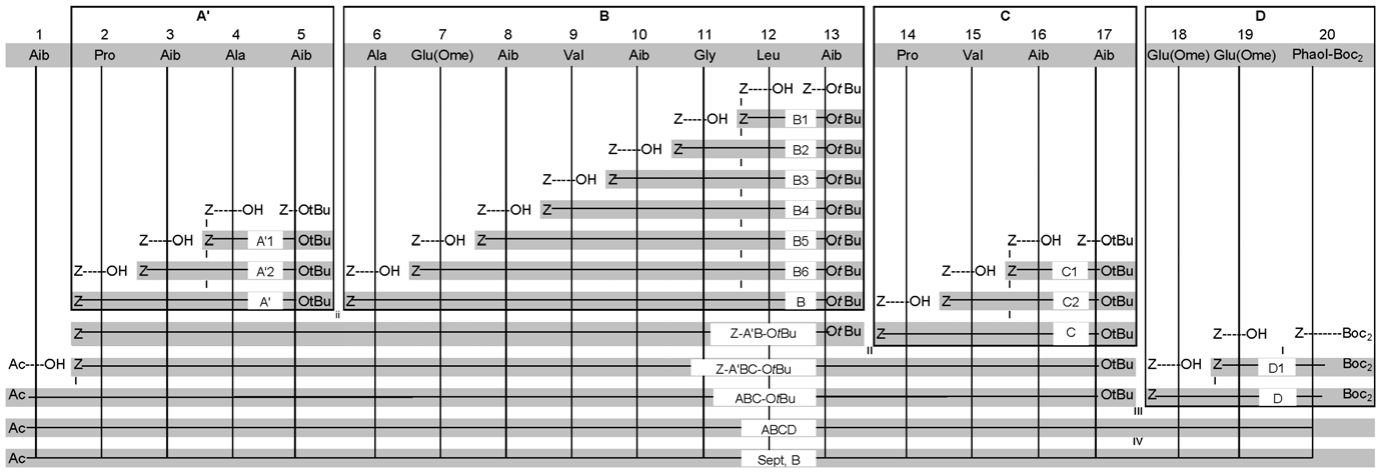
Synthetic strategy for the synthesis of Septocylindrin B with Z = Cbz. (i) Cbz-removal with H_2_, Pd-C and MeOH followed by coupling via EDC/HOAt. (ii) O*t*Bu-removal of the *C*-component with TFA/CH_2_Cl_2_ and Cbz-removal of the *N*-component with H_2_, Pd-C and MeOH, followed by coupling using EDC/HOAt. (iii) O*t*Bu-removal using ZnBr_2_ or TFA and Cbz-removal of the *N*-component with H_2_, Pd-C and MeOH, followed by coupling using EDC/HOAt, after which the peptide is Boc deprotected using ZnBr_2_. (iv) aminolysis.

The condensation approach for the completed fragments is depicted in Figure 3. Firstly, the *t*Bu group of the **A'** segment is cleaved off using TFA to couple it to H-**B**-O*t*Bu (obtained via Cbz hydrogenolysis of the corresponding segment), using HOAt and EDC, yielding 89% of Cbz-**A'B**-O*t*Bu. The *t*Bu group of this peptide was removed using TFA, after which it was coupled to the Cbz deprotected **C** segment, H-**C**-O*t*Bu, yielding 41% of Cbz-**A'BC**-O*t*Bu. This peptide was hydrogenolytically deprotected and coupled to Ac-Aib-OH, yielding 67% of **ABC**-O*t*Bu. This peptide was deprotected with ZnBr_2_, yielding 76% of **ABC**-OH. This molecule was coupled to the hydrogenolytically deprotected **D** segment, H-Glu(OMe)-Glu(OMe)-Phaol-(Boc)_2_, yielding 30% of **ABCD**. This peptide was then Boc deprotected with BiCl_3_ and transformed into Septocylindrin B by treatment with ammonia in methanol. The identity of the final peptaibol was confirmed by a high resolution ESI mass spectrum. No purification was needed as during the aminolysis reaction only Septocylindrin and methanol were formed. Thus, Septocylindrin B was tested for its antibacterial properties without any further purification. Interestingly, it shows comparable activity against *Staphylococcus aureus* as the natural Septocylindrin B (Table 1).

**Table 1:** Antibacterial activity (MIC, µM) of Septocylindrin B and selected analogues.^a^

	*Bacillus cereus*	*Bacillus subtilis*	*Enterococcus faecalis*	*Staphylococcus aureus*
**Septocylindrin B natural** b	NA^c^	NA	NA	8
**Septocylindrin B synthetic**	6.25	12.5	6.25	12.5
**ABCD-Phaol**	>50	>50	6	>50
**ABCD-Alaol**	>100	>100	>100	>100
**ABCD-Phe-ethanolamine**	100	>100	6	d
**ABCD-Phaol-N6**	>100	>100	25	>100
**ABCD-Phaol-N5O**	50	>100	1.5	d

a For HPLC traces of selected analogues see Figures S4, S5, S6, S7, S8, S9.

b Data from ref. [1].

c Not available.

d A reduced growth is clearly visible, but no MIC end point ≤100 µM.

### Synthesis of Analogues

**Figure 4:**
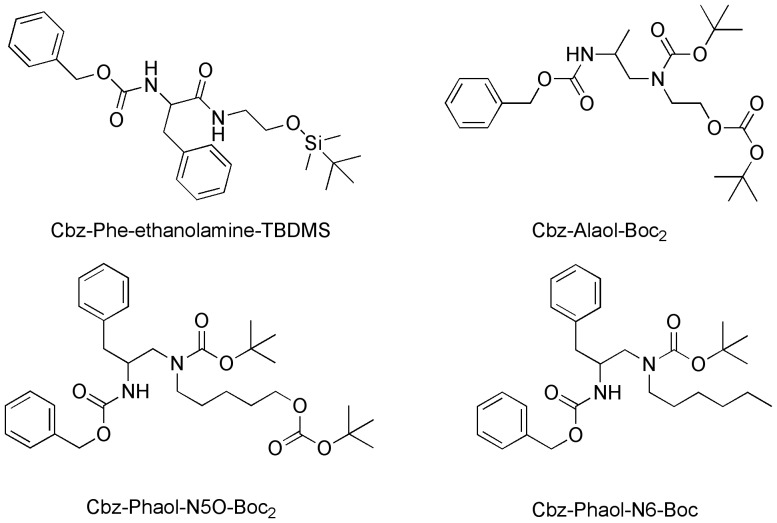
Analogues of Phaol, which are to be incorporated into the peptide chain.

After the successful synthesis of Septocylindrin B, several analogues were synthesized, characterized by the modified Phaol moieties described by Nelissen *et al.* [6] (Figure 4). Crisma *et al.* [11] investigated the influence of the replacement of Gln by Glu(OMe) in Alamethicin. They concluded that this substitution does not affect the backbone conformation and, in addition, they confirmed *via* patch-clamp experiments that it is also a functionally reliable model of its natural counterpart. Therefore we decided to test these newly synthesized Septocylindrin analogues, without converting the Glu(OMe) residues to glutamines.

The Septocylindrin analogues with -Alaol and -Phaol-N5O as terminal amino acid residues were synthesized using the same scheme as for Septocylindrin B: the first coupling to synthesize the **D** segment of **ABCD**-Phaol-N5O, yielded 99% of the dipeptide Cbz-Glu(OMe)-Phaol-N5O(Boc)_2_. The second glutamic acid was attached to yield 69% of Cbz-Glu(OMe)-Glu(OMe)-Phaol-N5O-Boc_2_. This **D** segment was coupled with **ABC**-OH, using PyBOP, in 40% yield. The peptide was deprotected using the BiCl_3_ mild procedure (to avoid the cleavage of the Aib-Pro tertiary amide bond). However, in this case only 12% of the fully deprotected peptide was isolated, while 75% of the mono Boc protected peptide was recovered.

The **D** segment of **ABCD**-Alaol, was synthesized in two successive deprotection and coupling reactions. The first and second coupling yielded 85% and 46% of Cbz-Glu(OMe)-Alaol(Boc)_2_ and Cbz-Glu(OMe)-Glu(OMe)-Alaol(Boc)_2_, respectively. This **D** segment was coupled with **ABC**-OH, using PyBOP, in 50% yield. Again, the BiCl_3_ deprotection step was not very effective: 29% of the fully deprotected peptide and 11% of the mono Boc protected peptide was isolated.

Two other peptides, containing the terminal -Phaol-N6 and -Phe-ethanolamine, were synthesized via a slightly modified procedure. Except for the first segment, which now included the Ac-Aib residue from the start (segment **A** instead of segment **A'**), all other segments were identical. Segment condensations were executed in a different order.

**Figure 5:**
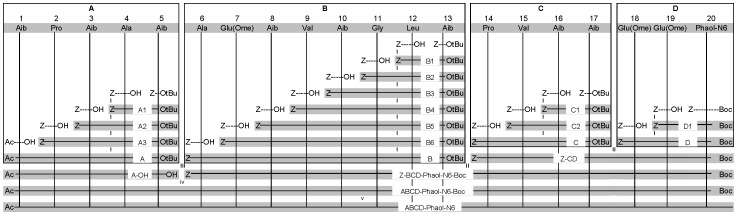
Synthetic strategy for the synthesis of ABCD-Phaol-N6 with Z = Cbz. (i) Cbz-removal with H_2_, Pd-C and MeOH followed by coupling via EDC/HOAt. (ii) O*t*Bu-removal of the *C*-component with TFA/CH_2_Cl_2_ and Cbz-removal of the *N*-component with H_2_, Pd-C and MeOH, followed by coupling using EDC/HOAt. (iii) O*t*Bu removal using ZnBr_2_ (iv) Cbz-removal of the *N*-component with H_2_, Pd-C and MeOH, followed by coupling via HOAt/EDC (v) Boc-deprotection using BiCl_3_.

The first coupling, to synthesize the **D** segment of **ABCD**-Phaol-N6 (Figure 5), yielded 77% of Cbz-Glu(OMe)-Phaol-N6-Boc. The coupling of the next glutamic acid yielded 84% of segment **D**. The following deprotection and segment condensation with Cbz-**C**-OH gave Cbz-**CD**-Phaol-N6-Boc in 79% yield. The next deprotection and condensation yielded 27% of Cbz-**BCD**-Phaol-N6. The last coupling was executed in a mixture of dichloromethane and dimethylsulfoxide (DMSO) (1∶1), instead of using only dichloromethane. This could help to overcome steric factors hindering the coupling. The condensation with segment **A** yielded 33% of **ABCD**-Phaol-N6-Boc. This peptide was deprotected using BiCl_3_, yielding only 7% of pure **ABCD**-N6.

**Figure 6:**
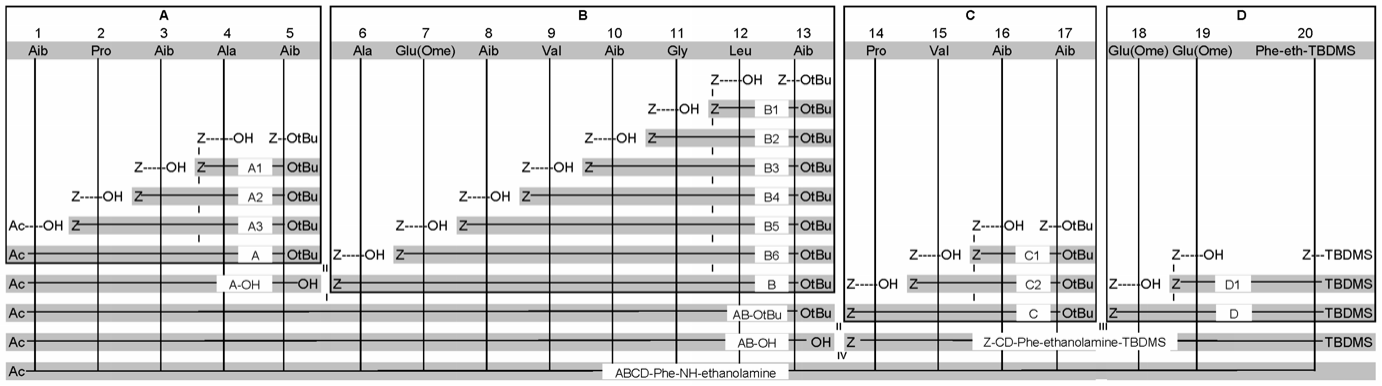
Synthetic strategy for the synthesis of ABCD-ethanolamine with Z = Cbz. (i) Cbz-removal with H_2_, Pd-C and MeOH followed by coupling via EDC/HOAt. (ii) O*t*Bu-removal with ZnBr_2_ .iii) O*t*Bu-removal of the *C*-component with TFA/CH_2_Cl_2_ and Cbz-removal of the *N*-component with H_2_, Pd-C and MeOH, followed by the coupling via EDC/HOAt. (iv) Cbz-removal with H_2_, Pd-C and MeOH followed by coupling via PyBOP, followed by deprotection using TBAF.

For the synthesis of **ABCD**-Phe-ethanolamine (See Figure 6), the **D** segment was synthesized in two coupling steps. The first attachment of Cbz-Glu(OMe)-OH yielded 88% of the dipeptide Cbz-Glu(OMe)-Phe-ethanolamine-TBDMS. The second deprotection and coupling yielded 80% of Cbz-Glu(OMe)-Glu(OMe)-Phe-ethanolamine-TBDMS. The **C** segment was coupled to the newly synthesized **D** segment in 55% yield. At the same time, **AB**-OtBu was synthesized in a yield of 67%, after which it was deprotected with ZnBr_2_ and coupled to H-**CD**-Phe-ethanolamine. The reaction mixture was then diluted in THF and cooled to −78°C. Next, TBAF was added and the reaction was stirred for 1 hour at room temperature, before work up. 43% of the deprotected **ABCD**-Phe-ethanolamine was obtained.

### Activity assays

Fluorescent dye leakage assays allowed verification whether the synthesized analogues were able to permeabilize membranes [4], [12]. Small unilamellar vesicles (SUV) of phosphatidylcholine (PC)∶cholesterol (Ch) (7∶3), encapsulating carboxyfluorescein (CF), were produced. To these SUVs increasing amounts of peptide were added. 20 minutes after addition, the fluorescence of the solution was measured. Being located in a limited space, the CF molecules in the liposomes display little fluorescence as auto-quenching occurs. If the integrity of the phospholipid membrane is disrupted, CF dilutes into the environment causing an increase in fluorescence intensity. This increase is related to the amount of CF released in the medium and, consequently, to the ability of the peptide to permeabilize membranes. After data collection, the SUVs were destroyed by adding Triton X-100 to the solution. The observed fluorescence was used as the reference value, according to the formula:
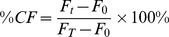
With: *F_0_* = intensity of fluorescence of the liposomes in absence of peptide.

*F_t_* = intensity of fluorescence at time t in the presence of peptide.

*F_T_* = intensity of fluorescence after liposome destruction by adding 50 µl 10% Triton X-100 in water.

**Figure 7:**
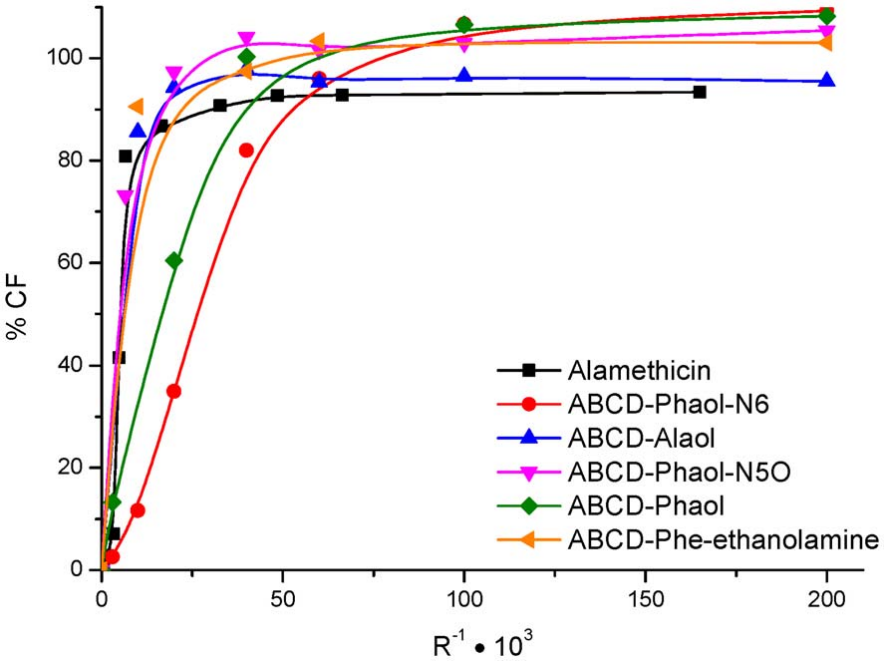
Release of CF from PC∶Ch (7∶3) liposomes after 20 minutes at different ratios R-1 = [peptide]/[lipid]. Alamethicin F50 is used as a reference.

The effect of the Phaol group and its analogues on membrane permeabilization was compared to that of Alamethicin. The results can be seen in Figure 7. They indicate that all synthesized analogues have the capacity to permeate a lipid bilayer. The peptides, still protected with one or two Boc-groups, were also tested (data not reported). They were less active, although not dramatically, as their unprotected derivative. Therefore, these results point to a possible role of the free amine and alcohol functions for the Septocylindrin interaction with membranes.

All these peptides were tested for their antibacterial activity using standard minimal inhibitory concentration (MIC) assays exactly as described in Mattheus *et al.* [13] Results are summarized in Table 1. The analogous peptides are more selective in comparison to the synthesized Septocylindrin B. Septocylindrin B shows activity against all four bacteria tested, whereas the analogues only show strong activity against *Enterococcus faecalis*, a Gram-positive bacterium resistant to many common antibiotics, including Vancomycin [14]. **ABCD**-Alaol showed no activity, which may indicate that the phenyl moiety in the terminal amino acid residue is necessary for activity. In view of (albeit low) **ABCD**-Phe-ethanolamine activity, the reduction of the amide function does not seem to be an absolute prerequisite for activity. **ABCD**-Phaol shows less activity compared to the native Septocylindrin B (fully deprotected). They are identical except for the glutamine residues, which are replaced with methyl protected glutamic acid residues in **ABCD**-Phaol. Although **ABCD**-Phaol should be a functionally reliable model of its natural counterpart [11], the difference in activity can only be explained by the different interaction with the membrane of the amino acid residue side chains. The amide interaction positively influences activity, so the conversion of Glu(OMe) to Gln improves the activity. The Boc-protected derivatives were also tested and showed no activity (data not shown), which again confirms the importance of the free amine and alcohol function.

## Conclusions

We were able to perform the total synthesis of the peptaibol Septocylindrin B and several of its analogues, avoiding the numerous drawbacks: the acid labile Aib-Pro linkage, the H-Aib-Pro- tendency to cyclize to diketopiperazine and the presence of an unusual moiety (Phaol). In particular, acid-free methods of −*t*Bu and Boc cleavage were employed in key steps: although the yields were low the syntheses were successful.

All these peptides were tested for their membrane perturbation properties by fluorescent dye leakage assay and antibacterial activity. The antibacterial tests show that the phenyl moiety is necessary for the activity, but not the reduced amide function. The conversion of Glu(OMe) to Gln improves the activity, thus calling for a role of the amide in its interaction with the membrane. Both in leakage assay and in antibacterial tests, the Boc-protected peptides show diminished activity in comparison to their unprotected derivatives. These results indicate the importance of the free amine and alcohol function for the interaction with the membrane.

## Supporting Information

Figure S1 **HPLC trace of ABCD-Phaol.** Mobile phase: methanol and a 0.1% solution of HCOOH in water. Run: 40% methanol to 100% methanol over 30 minutes, staying at 100% during 20 minutes.
(TIF)

Figure S2 **NMR of ABCD 25: ^1^H and ^13^C NMR, 600 MHz, DMSO, ppm.** The CO peaks could not be deduced from the HMBC spectrum.
(TIF)

Figure S3 **HPLC trace of Septocylindrin B.** Mobile phase: methanol and a 0.1% solution of HCOOH in water. Run: 80% methanol to 100% methanol over 20 minutes, staying at 100% methanol for 20 minutes.
(TIF)

Figure S4 **Mass spectrum of product peak of Septocylindrin B.**
(TIF)

Figure S5 **NMR of Cbz-BC^3^D^2^-Phaol-N6-Boc 30: ^1^H and ^DEPT^ NMR, 600 MHz, CDCl_3_, ppm.** The NMR peaks of following residues Phe interchangeable: 9 and 15; 7, 18 and 19.
(TIF)

Figure S6 **HPLC trace of ABCD-Phaol-N6.** Mobile phase: methanol and a 0.1% solution of HCOOH in water. Run: 40% methanol to 100% methanol over 30 minutes, staying at 100% during 20 minutes.
(TIF)

Figure S7 **HPLC trace of ABCD-Phe-ethanolamine.** Mobile phase: methanol and a 0.1% solution of HCOOH in water. Run: 40% methanol to 100% methanol over 30 minutes, staying at 100% during 20 minutes.
(TIF)

Figure S8 **HPLC trace of ABCD-Phaol-N5O.** Mobile phase: methanol and a 0.1% solution of HCOOH in water. Run: 40% methanol to 100% methanol over 30 minutes, staying at 100% during 20 minutes.
(TIF)

Figure S9 **HPLC trace of ABCD-Alaol.** Mobile phase: methanol and a 0.1% solution of HCOOH in water. Run: 40% methanol to 100% methanol over 30 minutes, staying at 100% during 20 minutes.
(TIF)

Supporting Information S1 **Experimental data and NMR-spectra of all new products.**
(DOCX)

## References

[1] Summers MY, Kong F, Feng X, Siegel MM, Janso JE, et al (2007) Septocylindrins A and B: Peptaibols Produced by the Terrestrial Fungus *Septocylindrium* sp. LL-Z1518. J Nat Prod 70: 391–396. 17288478 10.1021/np060571q

[2] Toniolo C, Brückner H (2009) Peptaibiotics. Wiley/VCH. 714p.

[3] Kastin AJ (2006) Handbook of biologically active peptides. Elsevier. 1595p.

[4] Stella L, Burattini M, Mazzuca C, Palleschi A, Venanzi M, et al (2007) Alamethicin Interaction with Lipid Membranes: A Spectroscopic Study on Synthetic Analogues. Chem Biodivers 4: 1299–1312. 17589867 10.1002/cbdv.200790111

[5] Kumazawa S, Kanda M, Aoyama H, Utagawa M, Kondo J, et al (1994) Structural elucidation of Aibellin, a new peptide antibiotic with efficiency enhancing activity on rumen fermentation. J Antibiot 47: 1136–1144. 7961164 10.7164/antibiotics.47.1136

[6] Nelissen J, Nuyts K, Dehaen W, De Borggraeve WM (2011) Synthesis of the orthogonally protected amino alcohol Phaol and analogs. J Pept Sci 17: 527–532. 21491546 10.1002/psc.1362

[7] Peggion C, Coin I, Toniolo C (2004) Total synthesis in solution of alamethicin F50/5 by an easily tunable segment condensation approach. Biopol pept sci 76: 485–493. 10.1002/bip.20161 15499566

[8] Theis C, Degenkolb T, Brückner H (2008) Studies on the Selective Trifluoroacetolytic Scission of Native Peptaibols and Model Peptides Using HPLC and ESI-CID-MS. Chem Biodivers 5: 2337–2335. 19035563 10.1002/cbdv.200890200

[9] Kaul R, Brouillette Y, Sajjadi Z, Hansford KA, Lubell WD (2004) Selective tert-Butyl Ester Deprotection in the Presence of Acid Labile Protecting Groups with Use of ZnBr_2_ . J Org Chem. 69: 6131–6133 b Wu Y-q, Limburg DC, Wilkinson DE, Vaal MJ, Hamilton GS (2004) A mild deprotection procedure for tert-butyl esters and tert-butyl ethers using ZnBr_2_ in methylene chloride. Tetrahedron Lett. 41: 2847–2849. 15373501 10.1021/jo0491206

[10] Navath RS, Pabbisetty KB, Hu LQ (2006) Chemoselective deprotection of N-Boc group in amino acids and peptides by bismuth(III) trichloride. Tetrahedron Lett 47: 389–393.

[11] Crisma M, Peggion C, Baldini C, MacLean EJ, Vedovato N, et al (2007) Crystal Structure of a Spin-Labeled, Channel-Forming Alamethicin Analogue. Angew Chem 46: 2047–2050. 17279588 10.1002/anie.200604417

[12] Peggion C, Jost M, Baldini C, Formaggio F, Toniolo C (2007) Total synthesis in solution of TOAC-labelled Alamethicin F50/5 Analogues. Chem Biodivers 4: 1183–1199 b Epand RF, Epand RM, Monaco V, Stoia S, Formaggio F, et al. (1999) The antimicrobial peptide Trichogin and its interaction with phospholipid membranes. Eur. J. Biochem. 266: 1021–1028. 10583397 10.1046/j.1432-1327.1999.00945.x

[13] Mattheus W, Gao L-J, Herdewijn P, Landuyt B, Verhaegen J, et al (2010) Isolation and purification of a new kalimantacin/batumin-related polyketide antibiotic and elucidation of its biosynthesis gene cluster. Chemistry and Biology 17: 149–159. 20189105 10.1016/j.chembiol.2010.01.014

[14] Amyes SGB (2007) Enterococci and streptococci. Int J Antimicrob Agents 29: S43–S52. 17659211 10.1016/S0924-8579(07)72177-5

