# Supplementary figures and images for: Total Synthesis of Septocylindrin B and C-Terminus Modified Analogues
Source: PLoS One. 2012 Dec 20;7(12):e51708. doi: 10.1371/journal.pone.0051708 (PMC3527430; doi:10.1371/journal.pone.0051708)

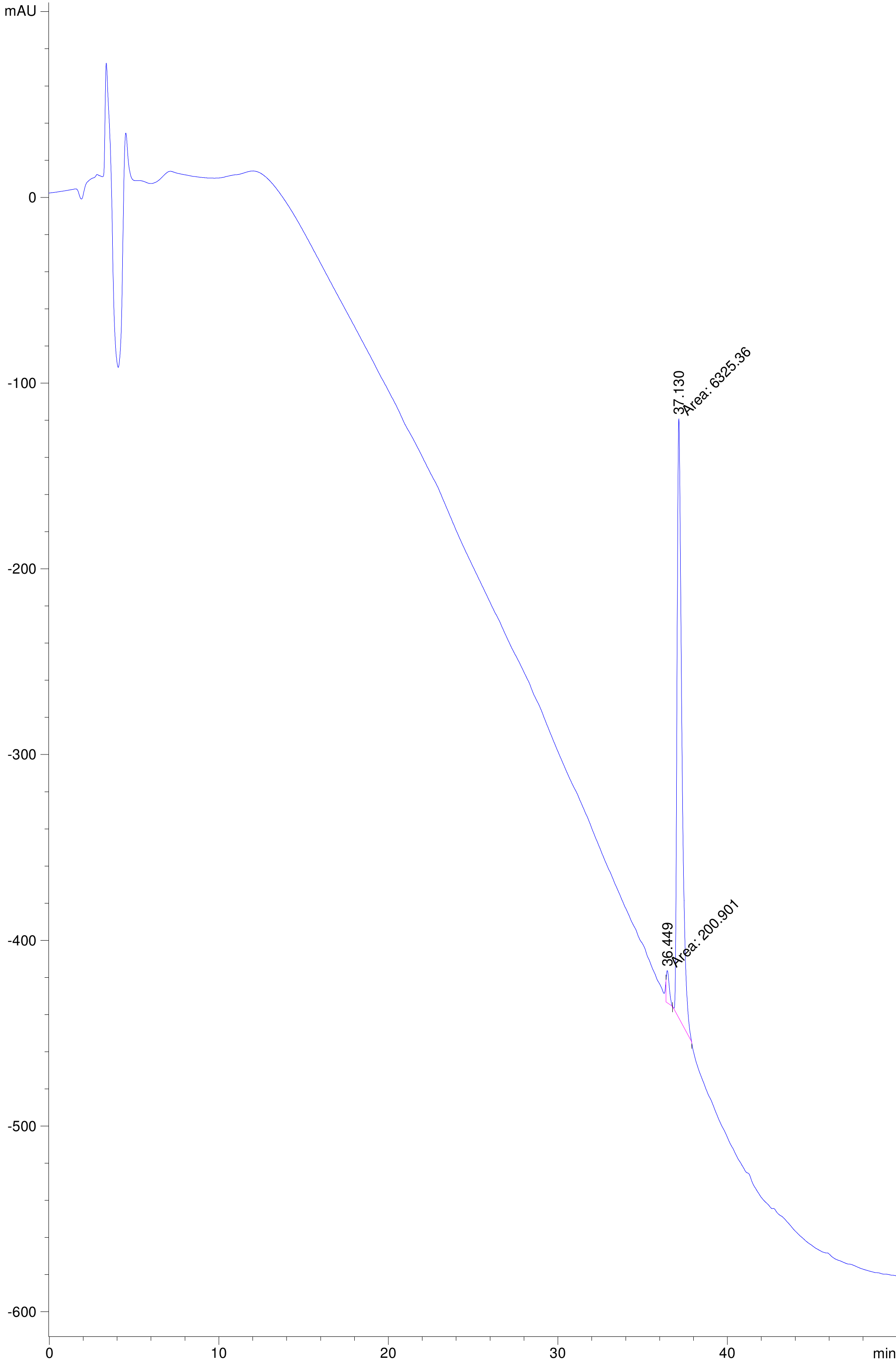

Supplement: Figure S1 — HPLC trace of ABCD-Phaol. Mobile phase: methanol and a 0.1% solution of HCOOH in water. Run: 40% methanol to 100% methanol over 30 minutes, staying at 100% during 20 minutes. (TIF) [file pone.0051708.s001.tif]

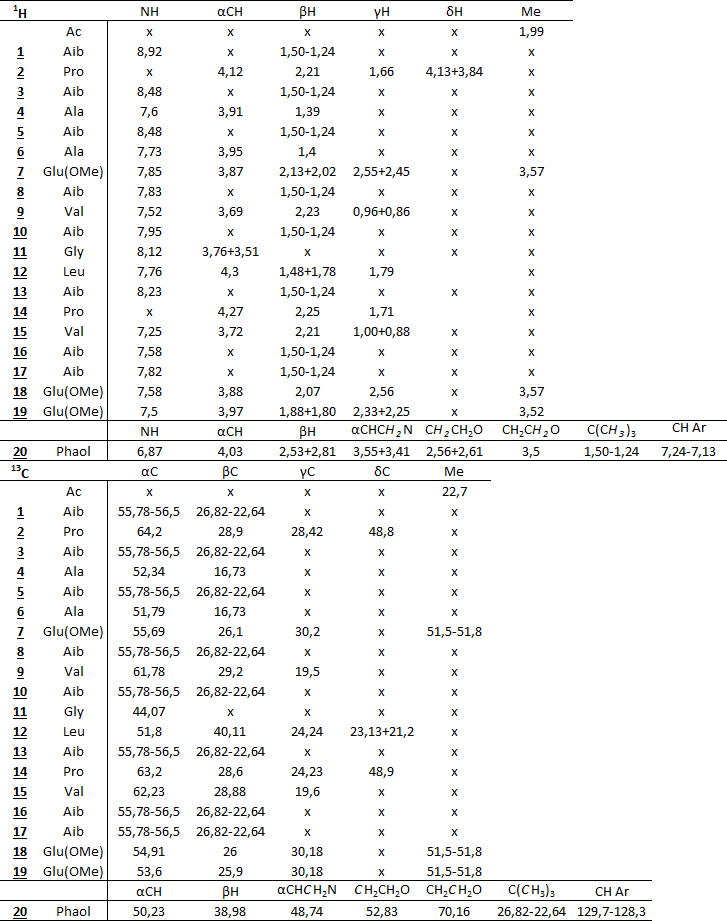

Supplement: Figure S2 — NMR of ABCD 25: 1H and 13C NMR, 600 MHz, DMSO, ppm. The CO peaks could not be deduced from the HMBC spectrum. (TIF) [file pone.0051708.s002.tif]

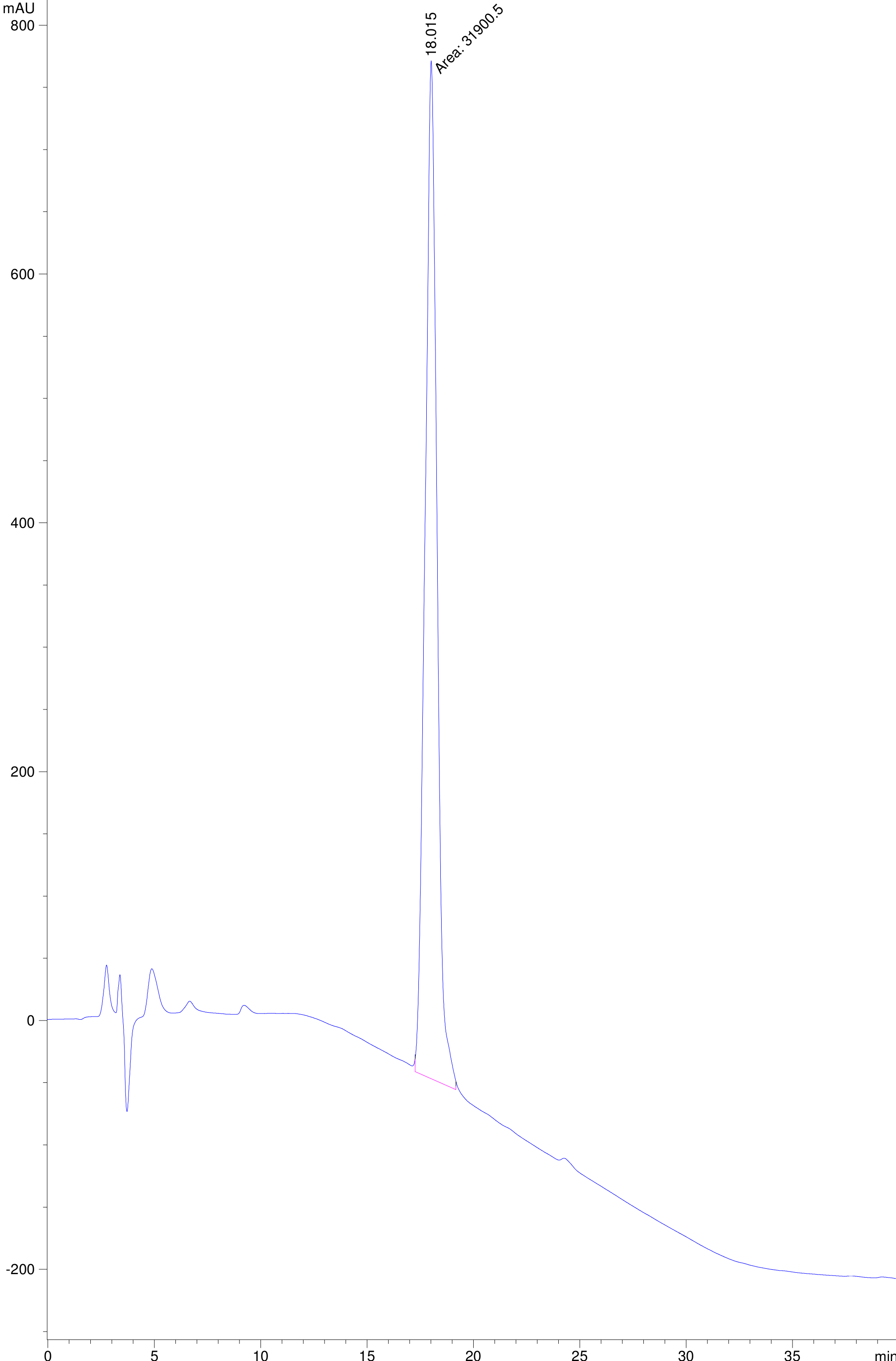

Supplement: Figure S3 — HPLC trace of Septocylindrin B. Mobile phase: methanol and a 0.1% solution of HCOOH in water. Run: 80% methanol to 100% methanol over 20 minutes, staying at 100% methanol for 20 minutes. (TIF) [file pone.0051708.s003.tif]

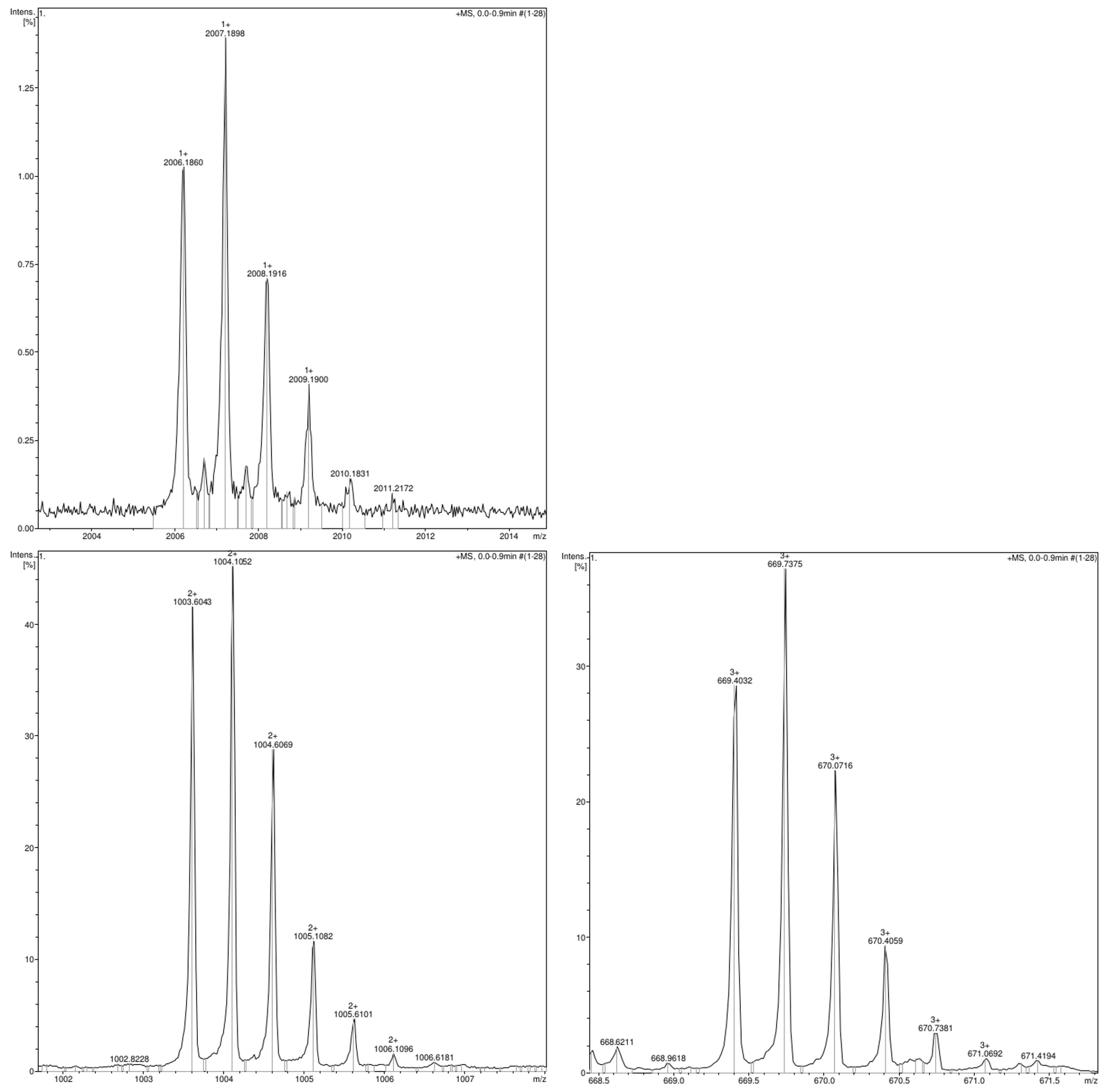

Supplement: Figure S4 — Mass spectrum of product peak of Septocylindrin B. (TIF) [file pone.0051708.s004.tif]

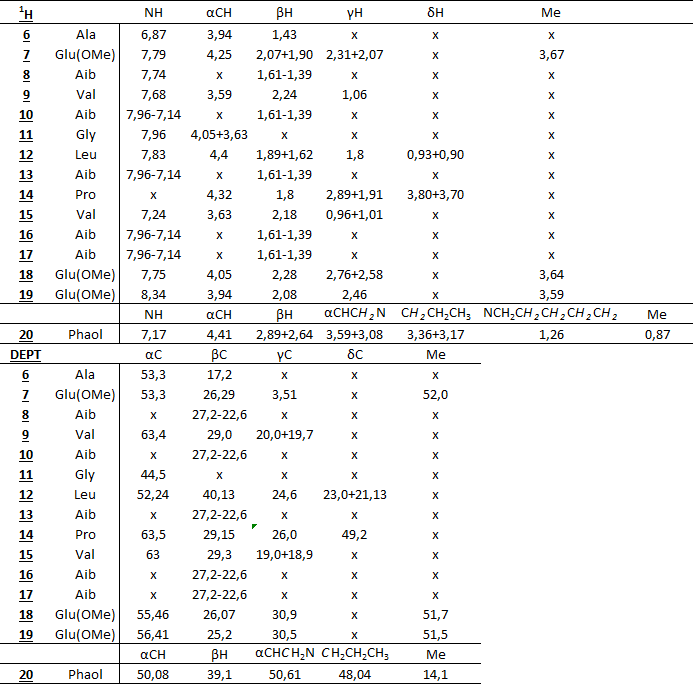

Supplement: Figure S5 — NMR of Cbz-BC3D2-Phaol-N6-Boc 30: 1H and DEPT NMR, 600 MHz, CDCl3, ppm. The NMR peaks of following residues Phe interchangeable: 9 and 15; 7, 18 and 19. (TIF) [file pone.0051708.s005.tif]

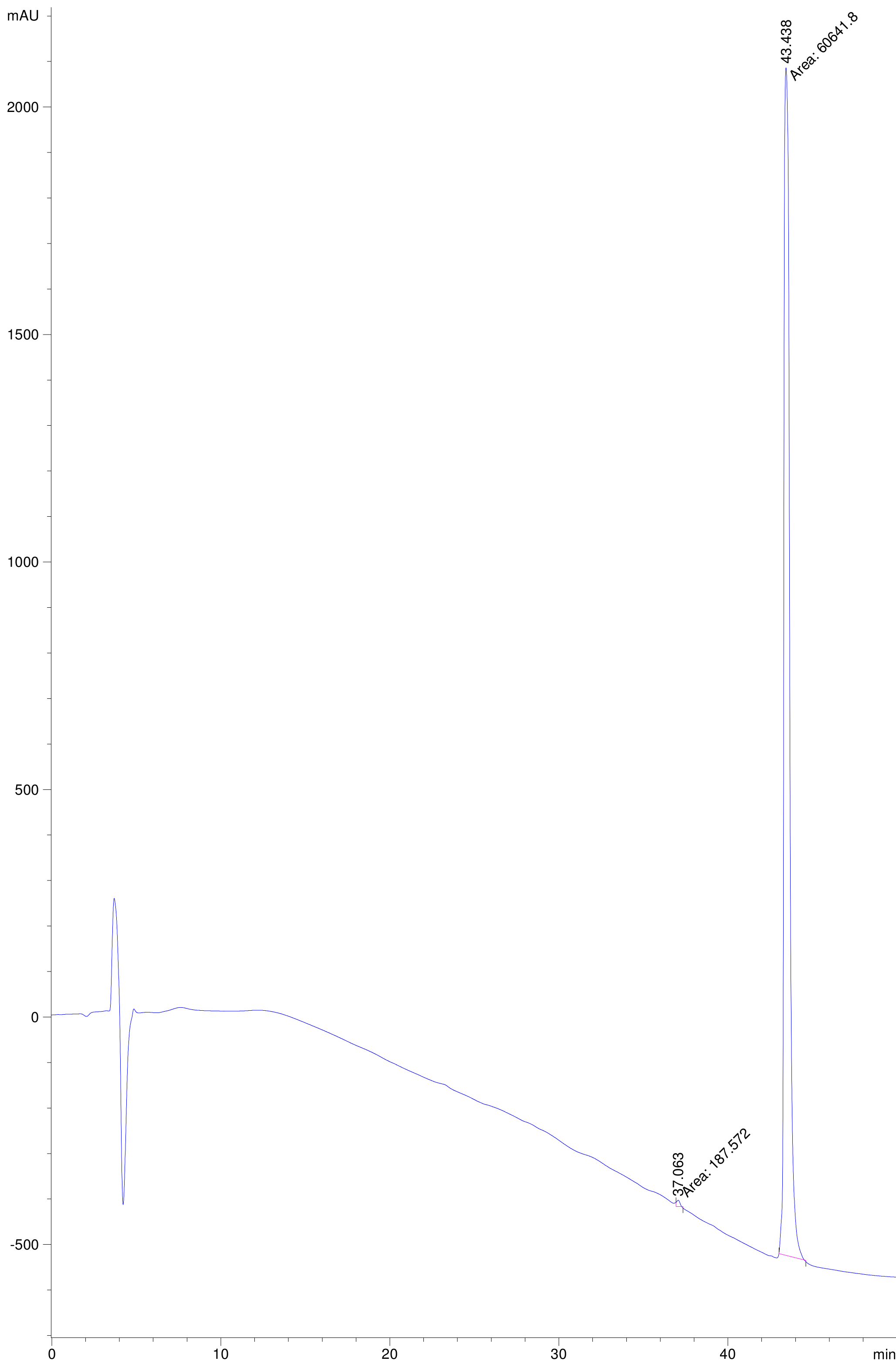

Supplement: Figure S6 — HPLC trace of ABCD-Phaol-N6. Mobile phase: methanol and a 0.1% solution of HCOOH in water. Run: 40% methanol to 100% methanol over 30 minutes, staying at 100% during 20 minutes. (TIF) [file pone.0051708.s006.tif]

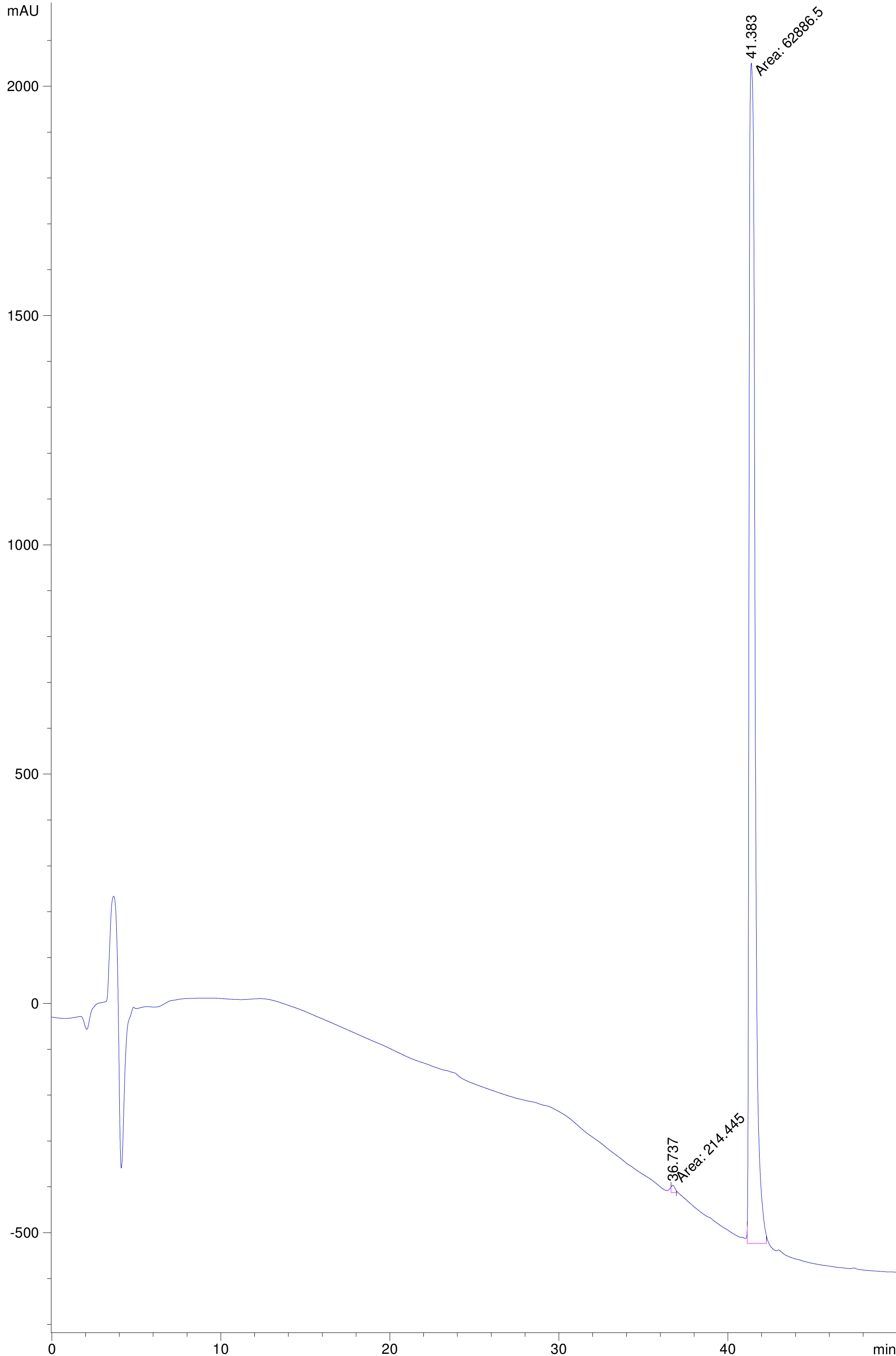

Supplement: Figure S7 — HPLC trace of ABCD-Phe-ethanolamine. Mobile phase: methanol and a 0.1% solution of HCOOH in water. Run: 40% methanol to 100% methanol over 30 minutes, staying at 100% during 20 minutes. (TIF) [file pone.0051708.s007.tif]

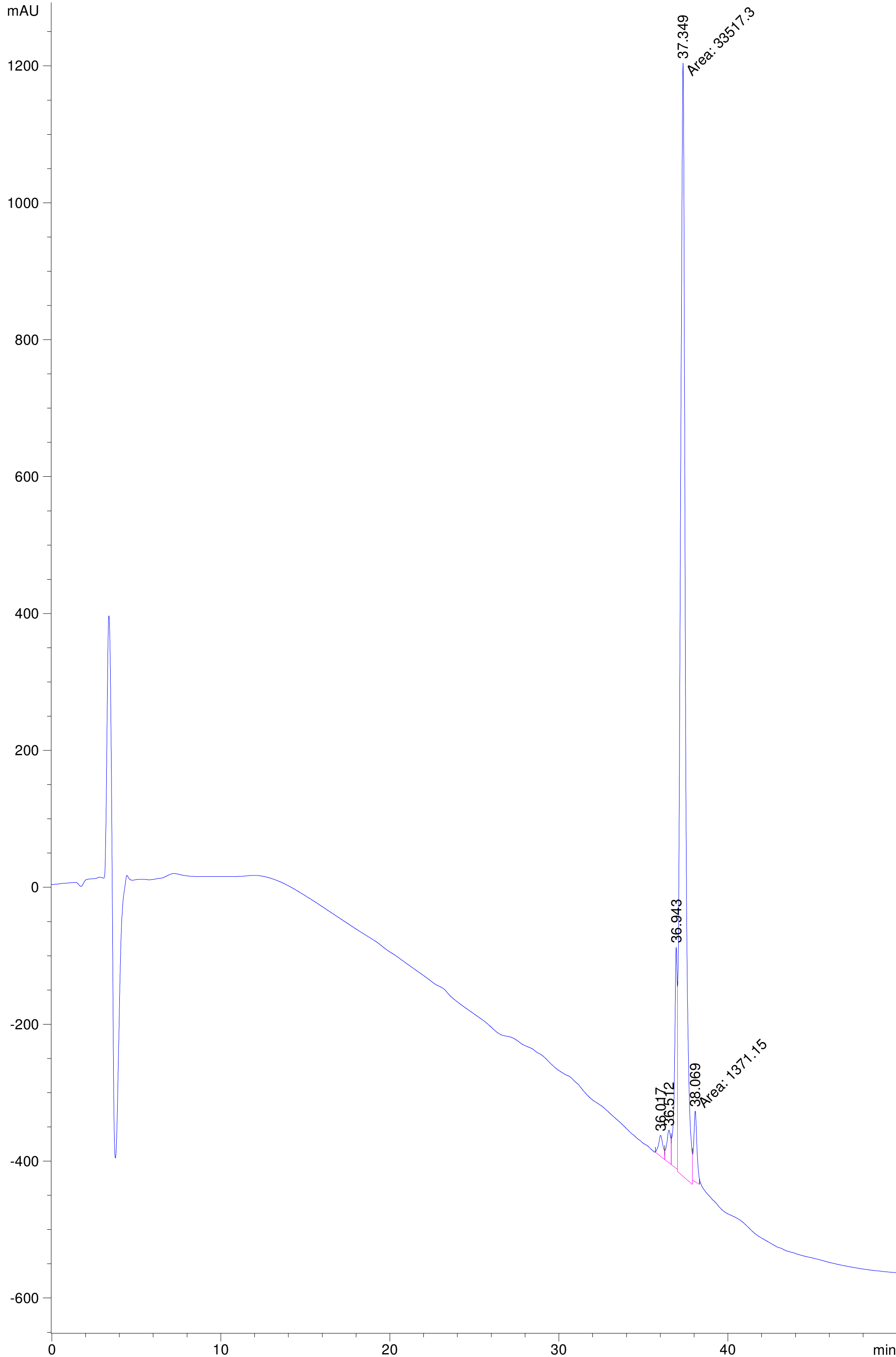

Supplement: Figure S8 — HPLC trace of ABCD-Phaol-N5O. Mobile phase: methanol and a 0.1% solution of HCOOH in water. Run: 40% methanol to 100% methanol over 30 minutes, staying at 100% during 20 minutes. (TIF) [file pone.0051708.s008.tif]

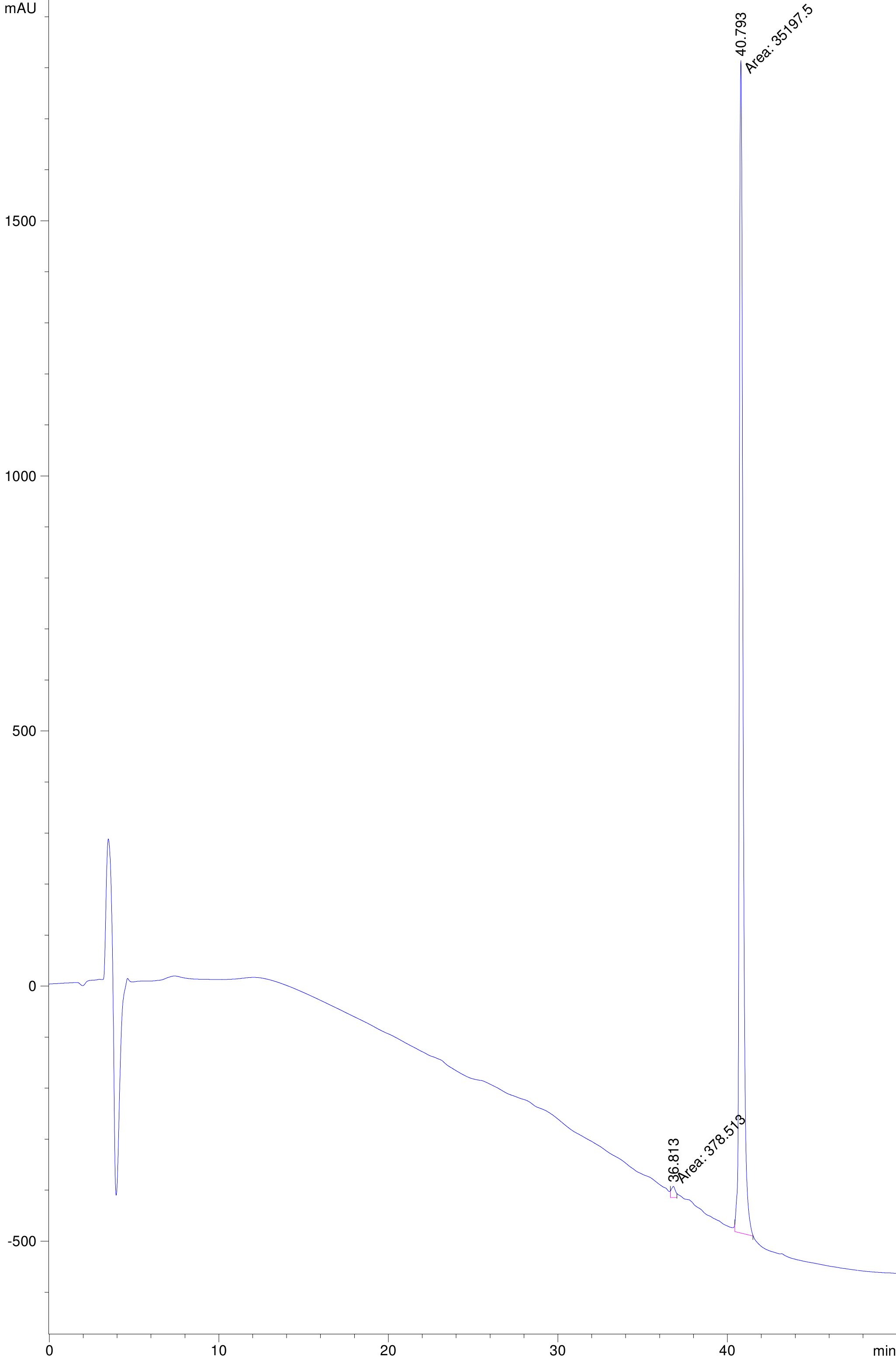

Supplement: Figure S9 — HPLC trace of ABCD-Alaol. Mobile phase: methanol and a 0.1% solution of HCOOH in water. Run: 40% methanol to 100% methanol over 30 minutes, staying at 100% during 20 minutes. (TIF) [file pone.0051708.s009.tif]
