# Supplementary material for: Total Synthesis of Septocylindrin B and C-Terminus Modified Analogues
Source: PLoS One. 2012 Dec 20;7(12):e51708. doi: 10.1371/journal.pone.0051708 (PMC3527430; doi:10.1371/journal.pone.0051708)
Supplement: Supporting Information S1 — Experimental data and NMR-spectra of all new products. (DOCX) [file pone.0051708.s010.docx]

Total synthesis of Septocylindrin B and C-terminus modified analogues

Jo Nelissen, [a] Koen Nuyts,[a] Marta De Zotti,[b] Rob Lavigne,[c] Chris Lamberigts,[c] and Wim M. De Borggraeve* [a]

[a] Molecular Design and Synthesis, Department of Chemistry, University of Leuven, Celestijnenlaan 200F, box 2404, BE-3001 Heverlee, Belgium

Fax: +32 16 32 79 90

E-mail: Wim.DeBorggraeve@chem.kuleuven.be

[b] Department of Chemistry, University of Padova, via Marzolo 1, 35131 Padova, Italy

[c] Laboratory of Gene Technology, Biosystems Department, University of Leuven, Kasteelpark Arenberg 21, box 2462, BE-3001 Heverlee, Belgium

Equipment used

Materials and Methods

The antibacterial assays were performed on *Bacillus cereus* (LMG 9610), *Bacillus subtilis* (ATCC 6633), *Enterococcus faecalis* (HC-1909-5) and *Staphylococcus aureus* (ATCC 6538). Alamethicin has been donated by the laboratory of prof. C. Toniolo and F. Formaggio. It has been synthesized according to [7]. The melting points were determined using a Reichert-Jung Thermovar or Electrothermal 9200 apparatus. The IR-spectra were recorded on a Bruker alpha-P. NMR spectra were recorded on a Bruker Avance 300 (300 MHz/75 MHz), Avance 400 (400 MHz/100 MHz) or Avance 600II+ (600 MHz/150 MHz) spectrometer with tetramethylsilane as internal standard. For the ESI-MS spectra, MW<2000: ESI-MS: Thermo Electron LCQ Advantage apparatus with Agilent 1100 pomp- and injection system coupled to a Xcalibur data analyzing software. MeOH or ACN were used as eluent. MW>2000: ESI-MS: Bruker Daltonics MicroTofQ mass spectrometer; ESI-MS: Bruker Daltonics Fourier Transform Ion Cyclotron Resonance (FT-ICR); MALDI-TOF: Bruker Daltonics ultraflex II TOF/TOF with aα-cyano-4-hydroxycinnamic acid (alpha) matrix. For purification, an MPLC Büchi Fraction collector C-660, Pump manager C-615, UV-photometer C-635, two Pump modules C-605 and Linseis D120S plotter were used. The flash silica gel used was Davisil® Chromatography Silica Medium, type LC 60A, 40-63 micron. For HPLC purifications, a Waters Delta 600 analytical/preparative system equipped with a Waters 996 Photo Diode Array detector. Preparative column: Alltech C18 Prevail 5 µm 150 nm x 22 mm. Waters alliance 2695 analytical system with a Waters 2487 dual λ absorbance detector. Column: Waters Xbridge BEH130 C18 3.5 µm 250 mm x 4.6 mm. Mobile phase: methanol or acetonitrile and water + 0.1% HCOOH.

Crystallizations were performed by dissolving the compound in a minimal amount of EtOAc and cooling the solution to 0°C. Heptane is slowly added under gentle shaking until a fine precipitate is formed. The solution is left standing in the refrigerator over night to allow crystallization. The crystals are filtered off and washed with cold heptane.

MeOH = methanol; EtOAc = ethyl acetate; DCM = dichloromethane; PE = petroleum ether; hept = heptane; EtOH = ethanol.; EDC.HCl = 1-(3-Dimethylaminopropyl)-3-ethylcarbodiimide hydrochloride; 1-hydroxybenzotriazole = HOBt; N-methyl morpholine = NMM; tetrahydrofuran = THF; sodium bis(2-methoxyethoxy)aluminum hydride = Red-Al; Di-tert-butyl dicarbonate = Boc2O; triethylamine = TEA; N-(Carbobenzyloxy)-L-phenylalanine = Cbz-Phe-OH; 1-hydroxy-7-azabenzotriazole = HOAt; N,N′-Dicyclohexylcarbodiimide = DCC; 4-(Dimethylamino)pyridine = DMAP; p-Toluenesulfonyl chloride = TsCl; N-(9-Fluorenylmethoxycarbonyloxy)succinimide = Fmoc-OSu; N-alpha-Benzyloxycarbonyl-L-glutamic acid gamma-methyl ester = Cbz-Glu(OMe)-OH; tert-butyl(chloro)dimethylsilane = TBDSCl; tetrabutylammonium fluoride =TBAF;

General procedures:

General procedure for coupling reactions:

If the amine is protected with a Cbz-group, the product is dissolved in MeOH, and 10 wt% of 10% Pd/C is added. This mixture is hydrogenated in the Parr apparatus until the deprotection is finished. Afterwards, the mixture is filtered over Celite and the solvent is evaporated.

1.3 equivalents of the acid are dissolved in anhydrous CH_2_Cl_2_ and cooled to 0 °C. 1.3 equivalents of 1-(3-dimethylaminopropyl)-3-ethylcarbodiimide hydrochloride (EDC.HCl) and 1.3 equivalents of 1-hydroxy-7-azabenzotriazole (HOAt) or 1-hydroxybenzotriazole (HOBt) are added at. This mixture is stirred for 15 min. after which it is added to the dissolved amine (1 eq.). *N-*methylmorpholine (NMM, 2 eq.) is added as base. The reaction is left stirring at room temperature and is followed *via* mass spectrometry. When finished, the mixture is washed with KHSO_4_ solution (5 %), NaHCO_3_ solution (5 %) and H_2_O, dried over Na_2_SO_4_ and the solvent is evaporated under reduced pressure. The product is purified either by column chromatography, crystallization or by precipitation.

General procedure for Cbz-protection:

To protect a free amine with a Cbz group, the unprotected amino acid is dissolved in water. Triethylamine (1 eq) is added, after which a solution of benzyloxycarbonyl-*N-*hydroxysuccinimide (1 eq) in acetonitrile is added. The pH is brought to 8-9 with triethylamine. The mixture is stirred overnight. For three days, benzyloxycarbonyl-*N-*hydroxysuccinimide (0.1 eq.) is added every day. The pH is kept basic with triethylamine. The mixture is extracted with diethyl ether to remove the excess benzyloxycarbonyl-*N-*hydroxysuccinimide. The water layer is acidified with K_2_SO_4_ and extracted with dichloromethane. The organic layer is washed with a KHSO_4_ solution (5%), a NaHCO_3_ solution (5%) and H_2_O, dried over Na_2_SO_4_ and evaporated under reduced pressure. The residue is purified by crystallization.

General method for *t-*butylation with isobutene:

Cbz-AA-OH is solved in dichloromethane (3 ml/mmol) in a pressure vial. At -78 °C isobutene (3 eq) is bubbled through and H_2_SO_4_ (0.01 ml/mmol) is added. The vial is closed and the mixture is stirred at room temperature for 3 days, after which it is cooled again to -78 °C and opened. The mixture is stirred, until the solvent level is constant (approx. 3 h). NaHCO_3_ solution (5 %) is added, and the mixture is extracted with dichloromethane. The organic phase is washed with KHSO_4_ solution (5%), NaHCO_3_ solution (5%)and H_2_O, dried over Na_2_SO_4_ and evaporated under reduced pressure. The residue is purified by crystallization.

General procedure for t-butyl deprotection, using ZnBr_2_:

The *t*-butyl protected product is dissolved in dry dichloromethane. ZnBr _2_ (10 eq) is added, and the mixture is allowed to stir at room temperature. The reaction is followed by mass spectrometry and every 3 days extra ZnBr_2_ (5 eq.) is added until the reaction is complete. The reaction is quenched with 0.2 N NaOH solution, the mixture is neutralized using KHSO_4_ solution. It is extracted with dichloromethane. The residue is purified by precipitation in cold diethyl ether.

General procedure for t-butyl deprotection, using TFA:

The *t*-butyl protected product is dissolved in dichloromethane. The mixture is cooled to 0 °C and TFA:DCM 1:1 is added (20 eq) is added, and the mixture is allowed to stir at room temperature. The reaction is followed by mass spectrometry. After the reaction is finished, the solvent and TFA is evaporated. Diethyl ether is added and evaporated until the peptide does not dissolve any more. The unprotected product is used without any further purification.

The known AA and fragments are characterized by ESI-MS and nmr. The new fragments are fully characterized.

For the NMR assignment, βCH_n_ in the reduced amino acid residues always refers to the βCH_n_ of the original AA. (With n= 1,2,3)

1. Cbz-Aib-OH

The product is synthesized using the general method for Cbz-protection described above.

H-Aib-OH (25 g, 0.24 mol); TEA (33.8 ml, 0.24 mol); Cbz-OSu (60.5 g, 0.24 mol); the residue is purified *via* crystallization.

**Yield:** 72% (41.28 g)

**^1^H NMR** (300 MHz, CDCl_3_, ppm): δ = 11.08 (br s, 1H, OH), 7.34 (s, 5H, Ph), 5.46 (br s, 0.8H, NH), 5.09 (s, 2H, C*H*_2_ Cbz), 1.57 (s, 6H, βH).

**^13^C NMR** (100 MHz, CDCl_3_, ppm): δ = 180.2 (*C*OOH), 155.7 (O*C*ONH), 136.6 (C Cbz), 129.4 + 128.9 + 128.5 (CH Cbz), 67.6 (*C*H_2_ Cbz), 56.8 (αC), 25.7 (βC).

**ESI:** 260 [M + H]^+^

1. Cbz-Leu-OH

The product is synthesized using the general method for Cbz-protection described above.

H-Leu-OH (10 g, 0.08 mol); TEA (11.2 ml, 0.08 mol); Cbz-OSu (19.9 g, 0.08 mol); the residue is purified *via* crystallization.

**Yield:** 95 % (19.2 g)

**^1^H NMR** (300 MHz, CDCl_3_, ppm): δ = 10.22 (br s, 1H, OH), 7.29 (s, 5H, Ph), 5.67 (d, *J* = 8 Hz, 0.8H, NH), 5.08 (s, 2H, C*H*_2_ Cbz), 4.43-4.36 (m, 1H, αH), 1.73-1.51 (m, 3H, β + γ H), 0.92 (s, 6H, δH)

**^13^C NMR** (75 MHz, CDCl_3_, ppm): δ = 177.9 (*C*OOH), 156.9 (O*C*ONH), 136.6 (C Cbz), 128.9 +, 128.6 + 128.5 (CH Cbz), 67.5 (*C*H_2_ Cbz), 52.8 (αC), 41.7 (βC), 25.1 + 23.3 + 22.0 (γ + δC)

**ESI:** 222 [M-COOH]^+^, 266 [M + H]^+^

1. Cbz-Aib-O*t*Bu

The product is synthesized using the *tert*-butylation method described above.

**Cbz-Aib-OH** 1 (1 g, 4.2 mmol); isobutene (4.8 ml, 51 mmol); H_2_SO_4_ (0.042 ml); the residue is purified *via* crystallization.

**Yield:** 80 % (0.98 g)

**^1^H NMR** (300 MHz, CDCl_3_, ppm): δ = 7.34 (m, 5H, Ph), 5.48 (br s, 0.8H, NH), 5.08 (s, 2H, C*H*_2_ Cbz), 1.64 (s, 6H, βH), 1.51 (s, 9H, C(C*H*_3_)_3_).

**^13^C NMR** (100 MHz, CDCl_3_, ppm): δ = 174.1 (*C*OO*^t^*Bu), 155.2 (O*C*ONH), 137.0 (C Cbz), 128.9 + 128.4 (CH Cbz), 81.9 (*C*(CH_3_)_3_), 66.6 (*C*H_2_ Cbz), 57.1 (αC), 28.2 (C(*C*H_3_)_3_), 25.3 (β*C*).

**ESI:** 317 [M + Na]^+^, 609 [2M + Na]^+^

1. Ac-Aib-OH

A mixture of H-Aib-OH (2 g, 20 mmol), acetic acid anhydride (5.9 ml, 3.2 eq, 62 mmol) and pyridine (5 ml, 3.2 eq, 62 mmol) is stirred at room temperature for 4 hours, after which it is reflux for one hour. Ice is added and the mixture is stirred for three hours. The solvent is evaporated and the residue is purified *via* crystallization.

**Yield:** 92 % (2.6 g)

**^1^H NMR** (300 MHz, DMSO, ppm): δ = 12.10 + 8.02 (2* s, 1H, NH + OH), 1.78 (C*H_3_*CO), 1.31 (s, 6H, βH).

**^13^C NMR** (75 MHz, DMSO, ppm): δ = 175.6 + 168.6 (*C*O), 54.6 (αC), 24.9 + 22.5 (β*C* + *C*H_3_CO).

**ESI:** 168.9 [M + Na]^+^, 314.1 [2M + Na]^+^

1. Cbz-Ala_4_-Aib_5_-O*t*Bu

The product is synthesized using the coupling method described above. Cbz deprotection is executed in dichloromethane, and the following evaporation is done at room temperature, because H-Aib-*t*Bu is volatile. The product is purified *via* column chromatography (EtOAc:PE 20:80).

**Cbz-Aib-O*t*Bu** 3 (5 g, 0.017 mol); Cbz-Ala-OH (5.13 g, 1.3 eq, 0.022 mol); EDC.HCl (4.22 g, 1.3 eq, 0.022 mol); HOAt (2.99 g, 1.3 eq, 0.022 mol); NMM (1.87 ml, 1 eq, 0.017 mol).

**Yield:** 89 % (5.5 g)

**^1^H NMR** (400 MHz, CDCl_3_, ppm): δ = 7.36-7.31 (m, 5H, Ph), 6.59 (br s, 0.8H, NH_5_), 5.32 (br s, 0.8H, NH_4_), 5.06 (s, 2H, C*H*_2_ Cbz), 4.18 (s, 1H, αH_4_), 1.52 (s, 6H, βH_5_), 1.45 (s, 9H, C(C*H*_3_)_3_), 1.37 (d, 6H, βH_4_).

**^13^C NMR** (100 MHz, CDCl_3_, ppm): δ = 173.9 (CO_5_), 171.9 (CO_4_), 156.6 (O*C*ONH), 136.6 (C Cbz), 129.0 + 128.7 + 128.3 (CH Cbz), 81.0 (*C*(CH_3_)_3_), 68.0 (*C*H_2_ Cbz), 62.2 (αC_5_), 48.60 (αC_4_), 28.2 (C(*C*H_3_)_3_), 25.3 (βC_5_), 17.7 (βC_4_).

**ESI:** 365.91 [M + H]^+^

1. Cbz-Aib_3_-Ala_4_-Aib_5_-O*t*Bu

The product is synthesized using the coupling method described above. The product is purified *via* column chromatography (EtOAc:PE 1:6).

**Cbz-Ala-Aib-O*t*Bu** 5 (1.71 g, 5.8 mmol); **Cbz-Aib-OH** 1 (1.80 g, 1.3 eq, 7.6 mmol); EDC.HCl (1.46 g, 1.3 eq, 7.6 mmol); HOAt (1.03 g, 1.3 eq, 7.6 mmol); NMM (1.18 ml, 2 eq, 7.6 mmol).

**Yield:** 84 % (2.16 g)

**^1^H NMR** (400 MHz, CDCl_3_, ppm): δ = 7.36-7.32 (m, 5H, Ph), 6.82 (br s, 0.8H, NH_5_), 6.55 (br s, 0.8H, NH_4_), 5.30 (br s, 0.8H, NH_3_), 5.09 (s, 2H, C*H*_2_ Cbz), 4.39 (m, 1H, αH_4_), 1.60-1.50 (m, 12H, βH_3, 5_), 1.45 (s, 9H, C(C*H*_3_)_3_), 1.33 (d, 3H, βH_4_).

**^13^C NMR** (75 MHz, CDCl_3_, ppm): δ = 173.3+ 173.4 + 171.0 (CO_3, 4, 5_), 155.2 (O*C*ONH), 136.0 (C Cbz), 128.6 + 128.3 + 128.1 (CH Cbz), 81.2 (*C*(CH_3_)_3_), 66.9 (*C*H_2_ Cbz), 56.9 + 56.7 (αC_3, 5_), 49.1 (αC_4_), 27.82 (C(*C*H_3_)_3_), 25.0 + 24.6 (β*C*_3, 5_), 18.0 (βC_4_).

**ESI:** 569 [M + Na]^+^

1. Cbz-Pro_2_-Aib_3_-Ala_4_-Aib_5_-O*t*Bu (Cbz-A’-O*t*Bu)

The product is synthesized using the coupling method described above. The product is purified *via* column chromatography (DCM:EtOH 30:1).

**Cbz-Aib-Ala-Aib-O*t*Bu** 6 (1.66 g, 3.7 mmol); Cbz-Pro-OH (1.2 g, 1.3 eq, 4.8 mmol); EDC.HCl (0.92 g, 1.3 eq, 4.8 mmol); HOAt (0.65 g, 1.3 eq, 4.8 mmol); NMM (0.41 ml, 1 eq, 3.7 mmol).

**Yield:** 85 % (1.71 g)

**^1^H NMR** (600 MHz, CDCl_3_, ppm): δ = 7.37-7.33 (m, 5H, Ph), 7.06 (d, *J* = 7 Hz, 0.8H, NH_4_), 7.03 + 6.53 2*(br s, 0.8H, NH_3, 5_), 5.19 + 5.14 2*(d, *J* = 12 Hz, 1H, C*H*_2_ Cbz), 3.35 (quintet, *J* = 7 Hz, 1H, αH_4_), 4.17 (t, *J* = 6 Hz, 1H, αH_2_), 3.60-3.54 (m, 2H, δH_2_), 2.21-2.15 + 2.13-2.09 + 2.03-1.99 + 1.95-1.95 4*(m, 1H, βH_2_ + γH_2_), 1.52 + 1.49 + 1.47 + 1.37 (s, 15H, βH_3, 4, 5_), 1.44 (s, 9H, C(C*H*_3_)_3_).

**^13^C NMR** (100 MHz, CDCl_3_, ppm): δ = 173.6 + 173.4 + 171.7 + 171.4 (CO_2, 3, 4, 5_), 156.2 (O*C*ONH), 136.2 (C Cbz), 128.6 + 128.3 + 128.0 (CH Cbz), 80.6 (*C*(CH_3_)_3_), 67.6 (*C*H_2_ Cbz), 61.8 (αC_2_), 57.0 + 56.4 (αC_3, 5_), 49.3 (αC_4_), 47.2 (δC_2_), 29.3 (βC_2_ + γC_2_), 27.8 (C(*C*H_3_)_3_), 30.9 + 26.3 + 24.7 + 24.6 (β*C*_3, 5_), 17.3 (βC_4_).

**ESI:** 569 [M + Na]^+^

1. Ac-Aib_1_-Pro_2_-Aib_3_-Ala_4_-Aib_5_-O*t*Bu (A-O*t*Bu)

The product is synthesized using the coupling method described above. The product is purified *via* column chromatography (DCM:EtOH 35:1).

**Cbz-Pro-Aib-Ala-Aib-O*t*Bu** 7 (3.43 g, 6.3 mmol); **Ac-Aib-OH** 4 (1.18 g, 1.3 eq, 8.2 mmol); EDC.HCl (1.57 g, 1.3 eq, 8.2 mmol); HOAt (1.12 g, 1.3 eq, 8.2 mmol); NMM (0.69 ml, 1 eq, 6.3 mmol).

**Yield:** 53 % (1.78 g)

**Melting point:** 190-195 °C

**IR** (cm^-1^)**:** 3331, 3275, 1731, 1652, 1620, 1543, 1530.

**^1^H NMR** (600 MHz, CDCl_3_, ppm): δ = 7.78 (s, 1H, NH_1_), 7.72-7.71 (m, 2H, NH_3, 4_), 7.37 (s, 1H, NH_5_), 4.26 (quintet, *J* = 7 Hz, 1H, αH_4_), 4.20 (dd, *J* = 8 Hz, 10 Hz, 1H, αH_2_), 3.98-3.95 + 3.31-3.26 2*(m, 1H, δH_2_), 2.40-2.36 + 1.75-1.68 2*(m, 1H, βH_2_), 2.06 (s, 4H, C*H*_3_CO + γH_2_), 1.95-1.85 (m, 1H, γH_2_), 1.59 (s, 3H, βH_3_), 1.49 (s, 3H, βH_1_), 1.47 (s, 6H, βH_3, 5_), 1.46 (s, 3H, βH_5_), 1.45 (d, *J* = 7 Hz, 3H, βH_4_), 1.41 (s, 12H, C(C*H*_3_)_3_ + βH_1_)

**^13^C NMR** (150 MHz, CDCl_3_, ppm): δ = 175.2 (CO_3_), 173.6 (CO_5_), 173.5 (CO_1_), 172.9 (CO_2_), 172.5 (CO_4_), 170.5 (CH_3_*C*O), 80.0 (*C*(CH_3_)_3_), 65.9 (αC_2_), 56.9 (αC_3_), 56.5 (αC_1_), 56.1 (αC_5_), 49.8 (αC_4_), 48.7 (δC_2_), 28.8 (βC_2_), 27.9 (C(*C*H_3_)_3_), 27.6 + 23.4 (βC_3_), 26.4 (γC_2_), 26.0 + 24.2 (βC_1_), 25.6 + 24.2 (βC_5_), 22.9 (*C*H_3_CO), 17.5 (βC_4_).

**ESI:** 562.5 [M + Na]^+^, 1101.2 [2M + Na]^+^

1. Ac-Aib_1_-Pro_2_-Aib_3_-Ala_4_-Aib_5_-OH (A-OH)

The product is synthesized using the *t*-butyl deprotection method using ZnBr_2_ described above.

**Ac-Aib_1_-Pro_2_-Aib_3_-Ala_4_-Aib_5_-O*t*Bu** 8 (0.1 g, 0.2 mmol), ZnBr_2_ (1 g, 20 eq, 2 mmol).

**Yield:** 88 % (0.79 g)

**Melting point**: 220-230 °C

**IR** (cm^-1^)**:** 3320, 3249, 3066, 1756, 1643, 1616, 1538.

**^1^H NMR** (400 MHz, DMSO, ppm): δ = 8.60 (s, 1H, NH_1_), 7.73 (s, 1H, NH_3_), 7.51 (d, *J* = 7 Hz), 7.35 (s, 1H, NH_5_), 4.13-4.07 (m, 1H, αH_4_), 4.02 (t, *J* = 7 Hz, 1H, αH_2_), 3.78-3.72 + 3.28-3.21 2*(m, 1H, δH_2_), 2.24-2.15 + 1.66-1.53 2*(m, 1H, βH_2_), 1.94 (s, 4H, C*H*_3_CO + γH_2_), 1.85-1.78 (m, 1H, γH_2_), 1.41 (s, 3H, βH_3_), 1.36 + 1.35 (s, 15H, βH_1, 3, 5_), 1.24 (d, *J* = 7 Hz, 3H, βH_4_).

**^13^C NMR** (100 MHz, DMSO, ppm): δ = 175.8 (CO_5_), 174.3 (CO_3_), 173.5 (CO_1_), 172.9 (CO_2_), 171.8 (CO_4_), 170.2 (CH_3_*C*O), 64.1 (αC_2_), 56.5 (αC_3_), 56.1 (αC_1_), 55.1 (αC_5_), 49.2 (δC_2_), 48.6 (αC_4_), 28.6 (βC_2_), 27.0 + 24.2 (βC_3_), 26.2 (γC_2_), 26.2 + 25.4 + 25.1 + 24.3 (βC_1, 5_), 22.7 (*C*H_3_CO), 17.6 (βC_4_).

**ESI:** 485 [M + H]^+^, 507 [M + Na]^+^, 990 [2M + Na]^+^

1. Cbz-Leu_12_-Aib_13_-O*t*Bu

The product is synthesized using the coupling method described above. The product is purified *via* column chromatography (EtOAc:PE 1:6).

**Cbz-Aib-O*t*Bu** 3 (5 g, 0.017 mol); **Cbz-Leu-OH** 2 (7.2 g, 1.3 eq, 0.022 mol); EDC.HCl (4.22 g, 1.3 eq, 0.022 mol); HOAt (2.99 g, 1.3 eq, 0.022 mol); NMM (1.87 ml, 1 eq, 0.017 mol).

**Yield:** 78 % (5.38 g)

**^1^H NMR** (400 MHz, CDCl_3_, ppm): δ = 7.35 (s, 5H, Ph), 6.59 (s, 0.8H, NH_13_), 5.16 (d, *J* = 8 Hz, 0.8H, NH_12_), 5.11 (s, 2H, C*H*_2_ Cbz), 4.12 (br s, 1H, αH_12_), 1.70-1.60 (m, 3H, β + γ H_12_), 1.51 (s, 6H, βH_13_), 1.45 (s, 9H, C(C*H*_3_)_3_), 0.95 + 0.93 2*(s, 3H, δH_12_).

**^13^C NMR** (75 MHz, CDCl_3_, ppm): δ = 173.5 (CO_13_), 171 (CO_12_), 156.1 (O*C*ONH), 136.3 (C Cbz), 128.5 + 128.2 + 128.0 (CH Cbz), 81.6 (*C*(CH_3_)_3_), 67.0 (*C*H_2_ Cbz), 56.9 (αC_13_), 53.5 (αC_12_), 41.7 (βC_12_), 27.8 (C(*C*H_3_)_3_), 24.7 + 24.4 + 24.3 + 22.9 + 22.0 (γ + δC_12_, βC_13_).

**ESI:** 351 [M-*t*Bu]^+^, 407 [M + H]^+^

1. Cbz-Gly_11_-Leu_12_-Aib_13_-O*t*Bu

The product is synthesized using the coupling method described above. The product is purified *via* column chromatography (DCM:EtOH 38:1).

**Cbz-Leu-Aib-O*t*Bu 10** (4.11 g, 10 mmol); Cbz-Gly-OH (2.99 g, 1.3 eq, 14 mmol; EDC.HCl (2.75 g, 1.3 eq, 14 mmol); HOAt (1.95 g, 1.3 eq, 14 mmol); NMM (1.21 ml, 1 eq, 10 mmol)

**Yield:** 76 % (3.5 g)

**^1^H NMR** (300 MHz, CDCl_3_, ppm): δ = 7.34 (s, 5H, Ph), 6.80 (s, 0.8H, NH_12, 13_), 5.70(s, 0.8H, NH_11_), 5.11 (s, 2H, C*H*_2_ Cbz), 4.47-4.42 (m, 1H, αH_12_), 3.97-3.82 (m, 2H, αH_11_), 1.70-1.54 (m, 3H, β + γ H_12_), 1.48 + 1.47 2*(s, 6H, βH_13_), 1.44 (s, 9H, C(C*H*_3_)_3_), 0.92 (s, 6H, δH_12_).

**^13^C NMR** (75 MHz, CDCl_3_, ppm): δ = 173.4 + 170.8 + 169.0 (CO_11, 12, 13_), 156.6 (O*C*ONH), 136.3 (C Cbz), 128.5 + 128.2 + 128.1 (CH Cbz), 81.6 (*C*(CH_3_)_3_), 67.2 (*C*H_2_ Cbz), 56.9 (αC_13_), 52.8 (αC_12_), 44.5 (αC_11_), 41.4 (βC_12_), 27.8 (C(*C*H_3_)_3_), 24.7 + 24.5 + 24.4 + 22.9 + 22.1 (γ + δC_12_, βC_13_).

**ESI:** 465 [M + H]^+^, 487 [M + Na]^+^

1. Cbz-Aib_10_-Gly_11_-Leu_12_-Aib_13_-O*t*Bu

The product is synthesized using the coupling method described above. The product is purified *via* column chromatography (DCM:EtOH 23:1).

**Cbz-Gly-Leu-Aib-O*t*Bu 11** (4.4 g, 9.5 mmol); **Cbz-Aib-OH** 1 (2.94 g, 1.3 eq, 12 mmol); EDC.HCl (2.38 g, 1.3 eq, 12 mmol); HOAt (1.69 g, 1.3 eq, 12 mmol); NMM (1.05 ml, 1 eq, 9.5 mmol).

**Yield:** 86 % (4.49 g)

**^1^H NMR** (300 MHz, CDCl_3_, ppm): δ = 7.49 (d, *J* = 7 Hz, 1H, NH_12_),7.34 (s, 5H, Ph), 7.21 (t, *J =* 5 Hz, 1H, NH_11_), 6.93 (s, 0.8H, NH_13_), 5.71 (s, 1H, NH_10_), 5.13 + 5.04 2*(d, *J* = 12 Hz, C*H*_2_ Cbz), 4.41 (dd, *J* = 9 Hz, 13 Hz, 1H, αH_12_), 3.99-3.84 (m, 2H, αH_11_), 1.83-1.63 (m, 3H, β + γH_12_), 1.51 + 1.49 + 1.48 + 1.46 4*(s, 3H, βH_13_), 1.41 (s, 9H, C(C*H*_3_)_3_), 0.91 + 0.86 2*(d, *J* = 6 Hz, 3H, δH_12_).

**^13^C NMR** (75 MHz, CDCl_3_, ppm): δ = 175.4 (CO_13_), 173.9 + 171.7 + 169.5 (CO_10, 11, 12_), 156.3 (O*C*ONH), 135.9 (C Cbz), 129.0 + 128.9 + 128.5 (CH Cbz), 81.2 (*C*(CH_3_)_3_), 67.7 (*C*H_2_ Cbz), 57.5 + 56.9 (αC_10, 13_), 52.3 (αC_12_), 44.2 (αC_11_), 40.2 (βC_12_), 28.4 (C(*C*H_3_)_3_), 26.3 + 25.3 + 25.1 + 24.9 + 23.6 + 21.6 + 14.6 (γ + δC_12_, βC_10, 13_).

**ESI:** 571 [M + Na]^+^

1. Cbz-Val_9_-Aib_10_-Gly_11_-Leu_12_-Aib_13_-O*t*Bu

The product is synthesized using the coupling method described above. The product is purified *via* crystallisation in Et_2_O/n-hexane.

**Cbz-Aib-Gly-Leu-Aib-O*t*Bu 12** (3.1 g, 5.7 mmol); Cbz-Val-OH (1.85 g, 1.3 eq, 7.4 mmol); EDC.HCl (1.41 g, 1.3 eq, 7.4 mmol); HOAt (1.01 g, 1.3 eq, 7.4 mmol); NMM (0.62 ml, 1 eq, 5.7 mmol)

**Yield:** 86 % (3.14 g)

**^1^H NMR** (600 MHz, CDCl_3_, ppm): δ = 7.62 (d, *J* = 8 Hz, 1H, NH_12_),7.35-7.29 (m, 6H, Ph + NH_11_), 7.05 + 7.00 2*(s, 0.8H, NH_10, 13_), 6.46 (s, 1H, NH_9_), 5.13 + 5.06 2*(d, *J* = 13 Hz, C*H*_2_ Cbz), 4.40-4.36 (m, 1H, αH_12_), 3.86 (dd, *J =* 6 Hz, 17Hz, 1H, αH_11_), 3.67-3.63 (m, 2H, αH_9, 11_), 2.15-2.09 (m, 1H, βH_9_)+ 1.86-1.80 (m, 1H, βH_12_), 1.72-1.69 (m, 3H, β + γH_12_), 1.49 + 1.46 + 1.43 + 1.35 4*(s, 3H, βH_10, 13_), 1.41 (s, 9H, C(C*H*_3_)_3_), 1.00 + 0.98 2*(d, *J* = 7 Hz, 3H, γH_9_), 0.91 + 0.86 2*(d, *J* = 6 Hz, 3H, δH_12_).

**^13^C NMR** (75 MHz, CDCl_3_, ppm): δ = 175.0 (CO_10 or 11_), 173.7 (CO_13_), 171.9 (CO_9_), 171.6 (CO_12_), 169.1 (CO_10 or 11_), 157.7 (O*C*ONH), 135.9 (C Cbz), 128.7 + 128.5 + 128.3 (CH Cbz), 80.7 (*C*(CH_3_)_3_), 67.5 (*C*H_2_ Cbz), 62.3 (αC_9_), 56.8 + 56.5 (αC_10, 13_), 52.0 (αC_12_), 43.6 (αC_11_), 39.9 (βC_12_), 29.3 (βC_9_), 27.8 (C(*C*H_3_)_3_), 25.8 + 24.6 + 24.4 (βC_10, 13_), 24.9 (γC_12_), 23.3 + 21.2 (δC_12_), 19.2 + 18.7 (γC_9_).

**ESI:** 671 [M + Na]^+^

1. Cbz-Aib_8_-Val_9_-Aib_10_-Gly_11_-Leu_12_-Aib_13_-O*t*Bu

The product is synthesized using the coupling method described above. The product is purified *via* MPLC: DCM:MeOH 8:2 🡪7:3.

**Cbz-Val-Aib-Gly-Leu-Aib-O*t*Bu 13** (3.1 g, 4.8 mmol); **Cbz-Aib-OH** 1 (1.48 g, 1.3 eq, 6.2 mmol); EDC.HCl (1.20 g, 1.3 eq, 6.2 mmol); HOAt (0.85 g, 1.3 eq, 6.2 mmol); NMM (0.53 ml, 1 eq, 4.8 mmol).

**Yield:** 77 % (2.73 g)

**^1^H NMR** (600 MHz, CDCl_3_, ppm): δ = 8.04-8.02 (m, 3H, NH_8, 11_), 7.91 (d, *J* = 8 Hz, 1H, NH_12_), 7.7 (d, *J* = 6 Hz, 1H, NH_9_), 7.65 (s, 1H, NH_13_), 7.39-7.29 (m, 6H, Ph), 5.14 + 5.08 2*(d, *J* = 13 Hz, C*H*_2_ Cbz), 4.37-4.33 (m, 1H, αH_12_), 3.90-3.85 (m, 2H, αH_9, 11_), 3.74 (dd, *J =* 6 Hz, 17Hz, 1H, αH_11_), 2.21-2.17 (m, 1H, βH_9_), 1.93-1.88 + 1.62-1.57 2*(m, 1H, βH_12_), 1.79-1.74 (m, 1H, γH_12_), 1.47 + 1.46 2*(s, 3H, βH_8_), 1.46 + 1.45 2*(s, 3H, βH_10_), 1.44 + 1.43 2*(s, 3H, βH_13_), 1.43 (s, 9H, C(C*H*_3_)_3_), 0.95-0.93 (m, 9H, γH_9_ + δH_12_), 0.89 (d, *J* = 6 Hz, 3H, δH_12_).

**^13^C NMR** (75 MHz, CDCl_3_, ppm): δ = 177.3 (CO_8_), 176.8 (CO_10_), 173.5 (CO_13_), 172.7 (CO_12_), 172.3 (CO_9_), 170.7 (CO_11_), 156.6 (O*C*ONH), 136.8 (C Cbz), 128.2 + 127.7 + 127.2 (CH Cbz), 80.4 (*C*(CH_3_)_3_), 66.3 (*C*H_2_ Cbz), 60.8 (αC_9_), 56.7 + 56.6 (αC_8, 10_), 56.2 (αC_13_), 51.9 (αC_12_), 43.2 (αC_11_), 40.0 (βC_12_), 29.0 (βC_9_), 26.8 (C(*C*H_3_)_3_), 24.9 + 24.8 (βC_8_), 24.3 + 24.2 (βC_13_), 23.5 + 23.4 (βC_10_), 23.7 (γC_12_), 22.4 + 19.8 (δC_12_), 18.1 + 17.8 (γC_9_).

**ESI:** 734 [M + H]^+^, 756 [M + Na]^+^

1. Cbz-Glu(OMe)_7_-Aib_8_-Val_9_-Aib_10_-Gly_11_-Leu_12_-Aib_13_-O*t*Bu

The product is synthesized using the coupling method described above. The product is purified via column chromatography (DCM:EtOH 15:1).

**Cbz-Aib-Val-Aib-Gly-Leu-Aib-O*t*Bu 14** (2.69 g, 3.7 mmol); Cbz-Glu(OMe)-OH (1.42 g, 1.3 eq, 4.7 mmol); EDC.HCl (0.92 g, 1.3 eq, 4.7 mmol); HOAt (0.65 g, 1.3 eq, 4.7 mmol); NMM (0.4 ml, 1 eq, 3.7 mmol).

**Yield:** 85 % (2.74 g)

**^1^H NMR** (400 MHz, CDCl_3_, ppm): δ = 7.76 (d, 1H, *J* = 8 Hz, NH_12_), 7.72-7.69 (m, 2H, NH_10, 11_), 7.37-7.30 (m, 5H, Ph), 7.28 (s, 1H, NH_8_), 7.24 (d, *J* = 5 Hz, 1H, NH_9_), 7.16 (s, 1H, NH_13_), 7.04 (d, *J* = 3 Hz, 1H, NH_7_), 5.16 + 5.11 2*(d, *J* = 12 Hz, C*H*_2_ Cbz), 4.40-4.34 (m, 1H, αH_12_), 4.06 +3.63 2*(dd, *J =* 6 Hz, 17Hz, 1H, αH_11_), 3.94-3.90 (m, 1H, αH_7_), 3.72-3.69 (m, 1H, αH_9_), 3.69 (s, 3H, Me_7_), 2.55 (m, 2H, γH_7_), 2.24-2.15 (m, 1H, βH_9_), 2.12-2.02 (m, 2H, βH_7_), 1.90-1.85 (m, 1H, βH_12_), 1.79-1.66 (m, 2H, βH_12_ + γH_12_), 1.55 + 1.48 2*(s, 3H, βH_10_), 1.50 + 1.37 2*(s, 3H, βH_8_), 1.47 + 1.45 2*(s, 3H, βH_13_), 1.41 (s, 9H, C(C*H*_3_)_3_), 1.00 + 0.97 2*(d, *J* = 7 Hz, 3H, γH_9_), 0.92 + 0.85 (d, *J* = 6 Hz, 3H, δH_12_).

**^13^C NMR** (100 MHz, CDCl_3_, ppm): δ = 176.6 (CO_10_), 176.0 (CO_8_), 173.7 (δC_7_), 173.6 (C_13_), 172.5 (CO_7_), 172.3 (C_9, 12_), 169.9 (CO_11_), 157.4 (O*C*ONH), 136.1 (C Cbz), 128.6 + 128.3 + 127.9 (CH Cbz), 80.3 (*C*(CH_3_)_3_), 67.2 (*C*H_2_ Cbz), 62.2 (αC_9_), 57.3 (αC_7_), 57.0 + 56.8 (αC_8, 10_), 56.3 (αC_13_), 52.4 (αC_12_), 52.0 (Me_7_), 44.1 (αC_11_), 39.9 (βC_12_), 30.6 (γC_7_), 29.1 (βC_9_), 27.8 (C(*C*H_3_)_3_), 27.2 (βC_8_), 26.7 (βC_10_), 25.5 (βC_7_), 25.4 + 24.2 (βC_13_), 24.5 (γC_12_), 23.5 + 23.4 (βC_8, 10_), 23.3 + 21.0 (δC_12_), 18.9 (γC_9_).

**ESI:** 877 [M + H]^+^, 898 [M + Na]^+^

1. Cbz-Ala_6_-Glu(OMe)_7_-Aib_8_-Val_9_-Aib_10_-Gly_11_-Leu_12_-Aib_13_-O*t*Bu (Cbz-B-O*t*Bu)

The product is synthesized using the coupling method described above. The product is purified via column chromatography (DCM:EtOH 16:1).

**Cbz-Glu(OMe)-Aib-Val-Aib-Gly-Leu-Aib-O*t*Bu 15** (2.7 g, 3.1 mmol); Cbz-Ala-OH (0.90 g, 1.3 eq, 4 mmol); EDC.HCl (0.77 g, 1.3 eq, 4 mmol); HOAt (0.55 g, 1.3 eq, 4 mmol); NMM (0.34 ml, 1 eq, 3.1 mmol).

**Yield:** 85 % (2.5 g)

**^1^H NMR** (600 MHz, CDCl_3_, ppm): δ = 8.19 (d, *J* = 3 Hz, 1H, NH_7_), 7.81 (d, *J* = 8 Hz, 1H, NH_12_), 7.76 (t, *J* = 6 Hz, 1H, NH_1_)_,_ 7.70 (s, 1H, NH_10_), 7.43 (s, 1H, NH_8_), 7.36-7.29 (m, 5H, Ph), 7.28 (s, 1H, NH_13_), 7.21 (d, *J* = 5 Hz, 1H, NH_9_), 6.96 (br s, 1H, NH_6_), 5.13 + 5.11 2*(d, *J* = 13 Hz, C*H*_2_ Cbz), 4.40-4.36 (m, 1H, αH_12_), 4.04 + 3.60 2*(dd, *J =* 6 Hz, 17Hz, 1H, αH_11_), 3.97-3.95 (m, 2H, αH_6,_ 7), 3.68-3.66 (m, 1H, αH_9_), 3.66 (s, 3H, Me_7_), 2.48-2.46 (m, 2H, γH_7_), 2.23-2.17 (m, 1H, βH_9_), 2.10-2.02 (m, 2H, βH_7_), 1.90-1.86 + 1.70-1.66 2*(m, 1H, βH_12_), 1.78-1.74 (m, 1H, γH_12_), 1.54 + 1.48 2*(s, 3H, βH_10_), 1.46 + 1.44 2*(s, 3H, βH_13_), 1.45 (s, 3H, βH_6_), 1.44 + 1.38 2*(s, 3H, βH_8_), 1.40 (s, 9H, C(C*H*_3_)_3_), 1.03 + 0.98 2*(d, *J* = 7 Hz, 3H, γH_9_), 0.89 + 0.84 (d, *J* = 7 Hz, 3H, δH_12_).

**^13^C NMR** (150 MHz, CDCl_3_, ppm): δ = 176.7 (CO_10_), 176.4 (CO_8_), 175.4 (CO_6_), 174.2 (δC_7_), 173.7 (C_13_), 172.6 (CO_9_), 172.4 (C_12_), 172.0 (CO_7_), 170.1 (CO_11_), 157.4 (O*C*ONH), 136.3 (C Cbz), 128.6 + 128.2 + 127.6 (CH Cbz), 80.2 (*C*(CH_3_)_3_), 67.1 (*C*H_2_ Cbz), 62.4 (αC_9_), 56.9 + 56.8 (αC_8, 10_), 56.3 (αC_13_), 56.1 (αC_7_), 53.1 (αC_6_), 52.5 (αC_12_), 52.1 (Me_7_), 44.1 (αC_11_), 40.0 (βC_12_), 30.5 (γC_7_), 29.2 (βC_9_), 27.8 (C(*C*H_3_)_3_), 27.0 (βC_8_), 26.7 (βC_10_), 25.1 (βC_7_), 25.5 + 24.5 (βC_13_), 24.2 (γC_12_), 23.4 + 23.3 (βC_8, 10_), 23.1 + 21.0 (γC_9_), 19.1 + 18.9 (δC_12_), 17.2 (βC_6_).

**ESI:** 948 [M + H]^+^, 970 [M + Na]^+^

1. Cbz-Aib_16_-Aib_17_-O*t*Bu

The product is synthesized using the general procedure for coupling reactions.

**Cbz-Aib-O*t*Bu** 3 (3 g, 10.2 mmol); Pd/C (0.6 g, 20 wt%); **Cbz-Aib-OH** 1 (3.15 g, 13.3 mmol); HOAt (1.81 g, 13.3 mmol); EDC.HCl (2.55 g, 13.3 mmol); NMM (1.13 ml, 10.2 mmol); the *N*-component is dissolved in dichloromethane instead of methanol; purification by crystallization.

**Yield:** 83% (3.21 g)

**Melting point**: 135 – 140 °C

**IR** (KBr, cm^-1^)**:** 3407, 3293, 1719, 1657, 1536, 1516.

**^1^H NMR** (200 MHz, CDCl_3_, ppm): δ = 7.35 (m, 5H, CH Cbz), 6.91 + 5.37 2*(s, 1H, NH), 5.10 (s, 2H, CH_2_ Cbz), 1.53 + 1.50 2*(s, 6H, βH), 1.45 (s, 9H, C(C*H*_3_)_3_).

**^13^C NMR** (75 MHz, CDCl_3_, ppm): δ = 173.9 + 173.1 (CO_16, 17_), 155.0 (CO Cbz), 136.4 (C Cbz), 128.5 + 128.1 + 128.0 (CH Cbz), 81.6 (*C*(CH_3_)_3_), 66.6 (CH_2_ Cbz), 56.9 + 56.8 (αC_16, 17_), 27.8 + 25.4 + 24.1 (C(*C*H_3_)_3_ + βC_16, 17_).

**ESI:** 402.0 [M + Na]^+^, 779.7 [2M + Na]^+^.

1. Cbz-Val_15_-Aib_16_-Aib_17_-O*t*Bu

The product is synthesized using the general procedure for coupling reactions.

**Cbz-Aib-Aib-O*t*Bu 17** (3.68 g, 9.73 mmol); Pd/C (0.37 g, 10 wt%); Cbz-Val-OH (3.17 g, 12.64 mmol), HOAt (1.72 g, 12.64 mmol), EDC.HCl (2.42 g, 12.64 mmol); NMM (1.07 ml; 9.73 mmol); purification by flash column chromatography (PE:EA 12:8), followed by crystallization.

**Yield:** 68% (3.15 g)

**Melting point**: 95–100 °C

**IR** (KBr, cm^-1^)**:** 3321, 3088, 3065, 3035, 3004, 2982, 2936, 2875, 1730, 1704, 1656, 1585, 1529.

**^1^H NMR** (300 MHz, CDCl_3_, ppm): δ = 7.35-7.34 (m, 5H, CH Cbz), 6.94 + 6.60 (2*s, 1H, NH_16, 17_), 5.36 (s, 1H, NH_1_*_5_*), 5.11 (s, 2H, CH_2_ Cbz), 3.90 (t, *J* = 7 Hz, 1H, αH_15_), 2.12-2.10 (m, 1H, βH_15_), 1.54 + 1.50 2*(s, 6H, βH*_16, 17_*), 1.45 (s, 9H, C(C*H*_3_)_3_), 0.98-0.92 (m, 6H, γH_15_).

**^13^C NMR** (75 MHz, CDCl_3_, ppm): δ = 173.8 + 173.0 + 170.6 (CO_15, 16, 17_), 156.5 (CO Cbz), 136.2 (C Cbz), 128.6 + 128.2 + 128.0 (CH Cbz), 81.5 (*C*(CH_3_)_3_), 67.1 (CH_2_ Cbz), 60.9 (αC_15_), 57.3 + 56.8 (αC_16, 17_), 31.0 (βC_15_), 27.8 (C(*C*H_3_)_3_), 25.1 + 24.6 + 24.3 + 24.2 (βC_16, 17_), 19.2 + 17.9 (γC_15_).

**ESI:** 478 [M + H]^+^

1. Cbz-Pro_14_-Val_15_-Aib_16_-Aib_17_-O*t*Bu (Cbz-C-O*t*Bu)

The product is synthesized using the general procedure for coupling reactions.

**Cbz-Val-Aib-Aib-O*t*Bu 18** (3.15 g, 6.60 mmol); Pd/C (0. 315g, 10 wt%); Cbz-Pro-OH (1.97 g, 8.57 mmol); HOBt (1.16 g, 8.57 mmol); EDC.HCl (1.64 g, 8.57 mmol); NMM (725 µl, 6.60 mmol); purification by flash column chromatography (PE:EA 1:2).

**Yield:** 88% (3.21 g)

**Melting point**: 120–125 °C

**IR** (KBr, cm^-1^)**:** 3486, 3421, 3375, 3341, 3058, 2982, 2970, 2955, 2931, 2882, 1731, 1691, 1657, 1643, 1533, 1509.

**^1^H NMR** (200 MHz, CDCl_3_, ppm): δ = 7.34 (s, 5H, CH Cbz), 6.98 (br s, 2H, *NH)*, 6.70 (br s, 1H, NH), 5.24-5.15 (m, 2H, CH_2_ Cbz), 4.38-4.35 (m, 1H, αH_14_), 4.17-4.02 (m, 1H, αH_15_), 3.54 (m, 2H, δH_14_), 2.19-1.91 (m, 5H, βH_15_ + βH_14_ + γH_14_), 1.51-1.44 (m, 22H, βH_16_ + βH_17_ + C(C*H*_3_)_3_), 0.91-0.80 (q, *J* = 7 Hz, 14 Hz, 6H, γH_15_).

**^13^C NMR** (75 MHz, CDCl_3_, ppm): δ = 173.9 + 173.1 + 172.4 + 170.3 (CO_14,15, 16, 17_), 156.2 (CO Cbz), 136.3 (C Cbz), 128.6 + 128.3 + 128.0 (CH Cbz), 81.0 (*C*(CH_3_)_3_), 67.5 (CH_2_ Cbz), 61.0 +60.4 (αC_14,15_), 57.2 + 56.8 (αC_16, 17_), 47.2 (δC_14_), 29.7 (βC_15_), 29.0 (βC_14_), 27.8 (C(*C*H_3_)_3_), 25.3 + 25.0 + 24.6 + 24.2 (βC_16, 17_), 24.7 (γC_14_), 19.3 + 17.6 (γC_15_).

**ESI:** 575 [M + H]^+^

1. Cbz-Glu(OMe)_18_-Glu(OMe)_19_-Phaol_20_-Boc_2_ (Cbz-D)

The product is synthesized using the general procedure for amino acid coupling reactions.

**Cbz-Glu(OMe)_19_-Phaol_20_-Boc_2_**

(0.69 g, 1.03 mmol); Pd/C (0.07 g, 10 wt%); Cbz-Glu(OMe)-OH (0.4 g, 1.33 mmol); HOAt (0.18 g, 1.33 mmol); EDC.HCl (0.25 g, 1.33 mmol); NMM (0.23 ml, 2.05 mmol); purification by MPLC heptane:ethylacetate 55:45 🡪 30:70.

**Yield:** 79% (0.66 g)

**Melting point**: 68 – 71 °C

**IR** (cm^-1^)**:** 3294, 2901, 1738, 1689, 1641, 1538.

**^1^H NMR** (400 MHz, CDCl_3_, ppm): δ = 7.37-7.18 (m, 11H, CH Ph + NH_19_), 6.93 (d, *J* = 7 Hz, 1H, NH_20_), 5.78 (br s, 1H, NH_18_) 5.164-5.08 (m, 2H, CH_2_ Cbz), 4.46-4.33 (m, 2H, αH_19, 20_), 4.22-4.09 (m, 3H, CH_2_O + αH_18_), 3.67 + 3.64 2*(s, 3H, OCH_3_), 3.59-3.53 + 3.20-3.17 2*(m, 1H, αHC*H_2_*N), 3.45-3.4 + 3.34-3.29 2*(m, 1H, C*H_2_*CH_2_O), 2.91-2.76 (m, 2H, βH_20_), 2.51-2.38 (m, 2H, γH_18_), 2.28 (m, 2H, γH_19_), 2.15-1.87 (m, 4H, βH_18, 19_), 1.48 + 1.45 2*(s, 9H, C(C*H*_3_)_3_).

**^13^C NMR** (100 MHz, CDCl_3_, ppm): δ = 173.8 + 173.7 (δCO_18, 19_), 171.0 (CO_18_), 170.4 (CO_19_), 156.5 + 153.3 (CO Cbz + CO Boc), 137.5 + 136.2 (C Ph), 129.2 + 128.5 + 128.4 + 128.2 + 128.0 + 126.5 (CH Ph), 82.2 + 80.6 (*C*(CH_3_)_3_), 67.1 (CH_2_ Cbz), 64.5 (*C*H_2_O), 54.5 (αC_18_), 52.6 (αC_19_), 51.9 + 51.7 2*(OCH_3_), 50.6 (αC_20_), 50.5 (αH*C*H_2_N), 46.7 (*C*H_2_CH_2_O), 38.6 βC_20_), 30.2 + 29.9 (γC_18, 19_), 28.3 + 27.7 (C(*C*H_3_)_3_), 27.8 (βC_18, 19_).

**ESI:** 838.3 [M + Na]^+^, 1651.7 [2M + Na]^+^

1. Cbz-Pro_2_-Aib_3_-Ala_4_-Aib_5_-Ala_6_-Glu(OMe)_7_-Aib_8_-Val_9_-Aib_10_-Gly_11_-Leu_12_-Aib_13_-OtBu (Cbz-A’B-O*t*Bu)

**Cbz-A‘-O*t*Bu** 7 is *t*Bu deprotected using the general procedure with TFA. The product is synthesized using the coupling method described above. The product is purified *via* MPLC: dichloromethane: MeOH 100:0 🡪94:6.

**Cbz-A’-O*t*Bu** 7 (1.88 g, 3.4 mmol); TFA (3.82 ml, 15 eq, 51.5 mmol);**Cbz-B-O*t*Bu 16** (3.9 g, 1.2 eq, 1.12 mmol); EDC.HCl (0.722 g, 1.1 eq, 3.8 mmol); HOAt (0.513 g, 1.1 eq, 0.513 mmol); NMM (0.75 ml, 2 eq, 6.86 mmol).

**Yield:** 86% (3.94 g)

Melting point: 115-119 °C

**IR** (cm^-1^)**:** 3309, 2981, 1736, 1650, 1530.

**^1^H NMR** (400 MHz, CDCl_3_, ppm): δ = 7.83-7.82 (m, 3H, NH_7,11,12_), 7.80 (s, 1H, NH_Aib_), 7.62 (s, 1H, NH_Aib_), 7.55 (d, *J* = 4 Hz, 1H, NH_4, 6_), 7.53 (s, 1H, NH_Aib_), 7.47 (d, *J* = 5 Hz, 1H, NH_4, 6_), 7.38-7.36 (m, 6H, Ph + NH_Aib_), 7.28 (d, *J* = 5 Hz, 0.9H, NH_9_), 7.10 (s, 1H, NH_Aib_), 5.23-5.18 (m, 2H, C*H*_2_ Cbz), 4.41-4.38 (m, 1H, αH_12_), 4.12 (t, *J* = 8 Hz, 1H, αH_2_), 4.07-4.04 (m, 1H, αH_4,6_), 4.02-4.00 (m, 1H, αH_11_), 3.96-3.91 (m, 2H, αH_4,6,7_), 3.69-3.61 (m, 7H, Me_7_ + αCH_9,11_ + δH_2_), 2.66-2.61 + 2.45-2.41 2*(m, 1H, βH_2_), 3.38-2.36 + 2.05-1.99 2*(m, 1H, γH_7_), 2.21 (m, 3H, βH_7,9_), 2.12-2.08 (m, 1H, γH_2_), 1.99-1.91 (m, 2H, γH_2_ + βH_12_), 1.83 (br s, 1H, γH_12_), 1.73-1.69 (γH_12_), 1.57-1.32 (m, 51H, βH_Aib,4,6_ + C(C*H*_3_)_3_), 1.08 + 0.99 2*(d, *J* = 7 Hz, 3H, γH_9_), 0.92 + 0.88 2*(d, *J* = 6 Hz, 3H, δH_12_).

**^13^C NMR** (100 MHz, CDCl_3_, ppm): δ = 176.9 + 176.5 + 176.3 + 176.0 + 174.9 + 173.9 + 173.4 + 173.1 + 173.0 + 172.9 + 172.2 + 170.2 (CO), 156.3 (O*C*ONH), 136.2 (C Cbz), 128.7 + 128.5 + 127.7 (CH Cbz), 80.1 (*C*(CH_3_)_3_), 67.7 (*C*H_2_ Cbz), 62.9 (αC_2,9_), 56.2 (αC_7_), 56.8 + 56.7 + 56.5 + 56.4 + 56.3 (αC_Aib_), 52.8 + 52.0 (αC_4,6_), 52.4 (αC_12_), 51.6 (Me_7_), 47.3 (δC_2_), 44.2 (αC_11_), 40.1 (βC_12_), 30.7 (βC_2_), 30.3 (γC_7_), 29.2 (βC_9_), 27.8 (C(*C*H_3_)_3_), 27.4 + 27.1 + 27.0 + 26.4 + 25.5 + 24.4 + 23.4 + 23.0 + 22.9 + 22.7 (βC_Aib_), 25.6 (βC_7_), 24.9 (γC_2_), 23.4 + 21.0 (δC_12_), 19.7 + 19.2 (γC_9_), 16.8 + 16.4 (βC_4,6_).

**ESI:** 1308.4 [M + Na]^+^

1. Cbz-Pro_2_-Aib_3_-Ala_4_-Aib_5_-Ala_6_-Glu(OMe)_7_-Aib_8_-Val_9_-Aib_10_-Gly_11_-Leu_12_-Aib_13_-Pro_14_-Val_15_-Aib_16_-Aib_17_-O*t*Bu (Cbz-A’BC-O*t*Bu)

**Cbz-A‘B-O*t*Bu 21** is *t*Bu deprotected using the general procedure with TFA. The product is synthesized using the coupling method described above. The product is purified *via* MPLC: dichloromethane: MeOH 100:0 🡪94:6.

**Cbz-A’B-O*t*Bu 21** (3.89 g, 3.03 mmol); TFA (3.38 ml, 15 eq, 45.5 mmol);**Cbz-C^3^-O*t*Bu 19** (1.91 g, 1.1 eq, 3.33 mmol); EDC.HCl (0.58 g, 1 eq, 3.03 mmol); HOAt (0.412 g, 1 eq, 3.03 mmol); NMM (0.6 ml, 2 eq, 6.06 mmol).

**Yield:** 41% (2.05 g)

**Melting point**: 122-126 °C

**IR** (cm^-1^)**:** 3525, 3315, 2981, 2361, 1737, 1651, 1530.

The NMR peak of residue 9 and 15 are interchangeable.

**^1^H NMR** (600 MHz, CDCl_3_, ppm): δ = 7.93 (t, *J* = 5 Hz, 1H, NH_11_), 7.90 + 7.83 + 7.61 + 7.29 + 6.93 + 6.58 (NH_Aib_), 7.79 (s, 1H, NH_12_), 7.78 (s, 1H, NH_9_), 7.77 (s, 1H, NH_7_), 7.47 (d, *J* = 4 Hz, 1H, NH_4_), 7.44 (s, 1H, NH_5_), 7.42-7.38 (m, 6H, CH Ph + NH_6_), 7.18 (d, *J* = 4 Hz, 1H, NH_15_), 5.27-5.17 (m, 2H, C*H*_2_ Cbz), 4.49-4.43 (m, 2H, αH_12,14_), 4.19-4.13 (m, 2H, αH_2,9_), 4.01-4.04 (m, 2H, αH_6,11_), 3.97-3.94 (m, 1H, αH_4_), 3.94-3.90 (m, 1H, αH_7_), 3.87-3.84 (m, 1H, δH_14_), 3.70-3.69 (m, 2H, δH_2_), 3.67 (s, 3H, Me_7_), 3.66-3.61 (m, 1H, αH_11_), 3.59-3.54 (m, 2H, αCH_15_ + δH_14_), 2.65-2.59 (m, 1H, γH_7_), 2.46-2.39 (m, 3H, βH_2, 9_ + γH_7_), 2.33 (m, 1H, βH_14_), 2.25-2.16 (m, 3H, βH_7,15_), 2.10-2.05 (m, 2H, γH_2,14_), 2.02-1.99 (m, 2H, βH_2_ + γH_2_), 1.89-1.80 (m, 4H, βH_12,14_ + γH_12,14_), 1.62-1.31 (m, 58H, βH_Aib,4,6,12_ + C(C*H*_3_)_3_), 1.11 + 1.01 2*(d, *J* = 7 Hz, 3H, γH_15_), 1.06 + 0.99 2*(d, *J* = 7 Hz, 3H, γH_9_), 0.90-0.88 (m, 6H, δH_12_)

**^13^C NMR** (150 MHz, CDCl_3_, ppm): δ = 177-170 (CO), 156.5 (O*C*ONH), 136.1 (C Cbz), 128.8 + 128.6 + 127.7 (CH Cbz), 80.0 (*C*(CH_3_)_3_), 67.8 (*C*H_2_ Cbz), 64.1 (αC_15_), 63.4 (αC_14_), 62.9 (αC_2_), 60.1 (αC_9_), 56.4 (αC_7_), 56.8 + 56.6 + 56.6 + 56.5 + 56.5 + 56.0 (αC_Aib_), 53.4 (αC_4_), 52.8 (αC_6_), 52.2 (αC_12_), 51.6 (Me_7_), 49.1 (δC_14_), 47.3 (δC_2_), 44.4 (αC_11_), 40.3 (βC_12_), 30.7(γC_7_), 30.2 (βC_2_), 29.4 (βC_14_), 29.2 + 29.2 (βC_9,15_), 27.9 (C(*C*H_3_)_3_), 27.4 + 27.1 + 27.0 + 26.5 + 26.2 + 25.9 + 25.3 + 24.7 + 24.5 + 23.6 + 23.0 + 22.8+ 22.7 (βC_Aib_), 26.2 (γC_14_), 25.6 (βC_7_), 24.9 (γC_2_), 24.4 (γH_12_), 23.2 + 21.1 (δC_12_), 20.2 + 19.2 (γC_15_), 19.4 + 18.6 (γC_9_), 16.7 + 16.5 (βC_4,6_).

**ESI:** 1674.8 [M + Na]^+^

1. Ac-Aib_1_-Pro_2_-Aib_3_-Ala_4_-Aib_5_-Ala_6_-Glu(OMe)_7_-Aib_8_-Val_9_-Aib_10_-Gly_11_-Leu_12_-Aib_13_-Pro_14_-Val_15_-Aib_16_-Aib_17_-OtBu (ABC-O*t*Bu)

The product is synthesized using the coupling method described above. The product is purified *via* MPLC: dichloromethane: MeOH 100:0 🡪92:8.

**Cbz-A’BC^3^-O*t*Bu 22** (2.01 g, 1.22 mmol); **Ac-Aib-OH** 4 (0.23 g, 1.3 eq, 1.6 mmol); EDC.HCl (0.3 g, 1.3 eq, 1.6 mmol); HOAt (0.22 g, 1.3 eq, 1.6 mmol); NMM (0.27 ml, 2 eq, 2.4 mmol).

**Yield:** 67% (1.34 g)

**Melting point**: 151-155 °C

**IR** (cm^-1^)**:** 3648, 3526, 3294, 2981, 2362, 2335, 1738, 1644, 1531.

The NMR peaks of following residues are interchangeable: 2 and 14; 4 and 6; 9 and 15.

**^1^H NMR** (600 MHz, CDCl_3_, ppm): δ = 8.17 (s, 1H, NH_1_), 8.11 + 7.97 + 7.66 + 7.42 (NH_Aib_), 8.04 (br s, 1H, NH_11_), 7.87-7.84 (m, 3H, NH_7,12,Aib_), 7.80 (d, *J* = 7 Hz, 1H, NH_6_), 7.60 (br s, 2H, NH_9, Aib_), 7.44 (d, *J* = 5 Hz, 1H, NH_4_), 7.31 (d, *J* = 5 Hz, 1H, NH_15_), 6.99(s, 1H, NH_8_), 4.89-4.46 (αH_12_), 4.42 (t, *J* = 8 Hz, 1H, αH_2_), 4.20 ((t, *J* = 8 Hz, 1H, αH_14_), 4.06-3.97 (m, 5H, αH_9,11,4,6_ + δH_14_), 3.88 (m, 2H, αH_7_ + δH_2_), 3.64 (m, 5H, αH_11_ + δH_2_ + Me_7_), 3.52-3.49 (m, 1H, αH_15_), 3.40-3.37 (m, δH_14_), 2.72-2.67 (m, 1H, γH_7_),2.44-2.37 (m, 3H, βH_9,14_ + γH_7_), 2.31-2.29 (m, 2H, βH_2,7_), 2.22-2.17 (m, 2H, βH_7,15_), 2.13 (s, 3H, Me_Ac_),2.11-2.07 (m, 2H, γH_2,14_), 1.98-1.94 (m, 1H, γH_14_), 1.90 (m, 3H, βH_12_ + γH_2,12_), 1.83-1.79 (m, 1H, βH_2_), 1.78-1.73 (m, 1H, βH_14_), 1.60-1.44 (m, 64H, βH_Aib,4,6,12_ + C(C*H*_3_)_3_), 1.12 (d, *J* = 6 Hz, 3H, γH_15_), 1.08 (d, *J* = 7 Hz, 3H, γH_9_), 1.00 (d, *J* = 6 Hz, 6H, γH_9,15_), 0.89 (d, *J* = 6 Hz, 6H, δH_12_).

**^13^C NMR** (150 MHz, CDCl_3_, ppm): δ = 177-72 (CO), 173.7 (CO_7_), 171.0 (CO_Ac_), 170.6 (CO_11_) 80.1 (*C*(CH_3_)_3_), 64.6 (αC_15_), 63.4 (αC_14_), 63.4 (αC_2_), 61.0 (αC_9_), 56.7 (αC_7_), 56.7 + 56.6 + 56.5 + 56.4 + 563 + 56.1 (αC_Aib_), 52.8 (αC_4_), 52.4 (αC_6_), 52.2 (αC_12_), 51.5 (Me_7_), 49.2 (δC_2_), 49.0 (δC_14_), 44.5 (αC_11_), 40.5 (βC_12_), 30.8 (γC_7_), 29.4 (βC_2_),29.3 + 29.2 (βC_9,15_), 28.9 (βC_14_), 27.9 (C(*C*H_3_)_3_), 27.1 + 26.9 + 26.4 + 26.2 + 26.0 + 25.4 + 24.4 + 24.1 + 24.0 + 23.5 + 22.9 + 22.7 + 22.7 + 22.6 (βC_Aib_), 26.4 (γC_14_),26.2 (γC_2_), 25.6 (βC_7_),24.5 (γH_12_),22.9 + 21.2 (δC_12_),20.5 (γC_15_), 19.4 + 19.3 (γC_9,15_), 19.1 (γC_9_), 16.8 + 16.6 (βC_4,6_).

**ESI:** 1667.9 [M + Na]^+^

1. Ac-Aib_1_-Pro_2_-Aib_3_-Ala_4_-Aib_5_-Ala_6_-Glu(OMe)_7_-Aib_8_-Val_9_-Aib_10_-Gly_11_-Leu_12_-Aib_13_-Pro_14_-Val_15_-Aib_16_-Aib_17_-Glu(OMe)_18_-Glu(OMe)_19_-Phaol_20_-Boc_2_ (ABCD-Boc_2_)

The product is synthesized using the general procedure for coupling reactions.

**ABC-O*t*Bu 23**  is *t*butyl deprotected using the deprotection method using TFA described above.

**ABC-OH** (0.15 g, 1 eq, 0.094 mmol); **Cbz-D 20** (0.093 g, 1.2 eq, 0.11 mmol); PyBOP (0.054 g, 1.1 eq, 0.1 mmol); NMM (0.02 ml, 2 eq, 0.19 mmol); purification by HPLC.

**Yield:** Strategy 3: 22% (0013 g)

Strategy 4: 30 % (0.064 g)

**ESI:** 2152.234 [M + H]^+^, 2174.117 [M + Na]^+^

1. Ac-Aib_1_-Pro_2_-Aib_3_-Ala_4_-Aib_5_-Ala_6_-Glu(OMe)_7_-Aib_8_-Val_9_-Aib_10_-Gly_11_-Leu_12_-Aib_13_-Pro_14_-Val_15_-Aib_16_-Aib_17_-Glu(OMe)_18_-Glu(OMe)_19_-Phaol_20_ (ABCD)

**ABCD-Boc_2_ 24** (0.0126 g, 1 eq, 0.0056 mmol) is dissolved in dry dichloromethane. 5 eq. of BiCl_3_ (0.009 g, 5 eq, 0.028 mmol) are added. The reaction is stirred overnight at reflux temperature. Water is added and the pH is made neutral. The mixture is extracted with dichloromethane. Purification is done by HPLC (see Figure S1).

**Yield**: 0.004 g (35 %)

**ESI**: 1037.5726 [M + Na + H]^2+^, 10455548 [M + K+H]^2+^, 2052.1135 [M + H]^+^

The NMR peaks of following residues are interchangeable: 7, 18 and 19; 9 and 15, NH_Aib_. The carbon spectrum is deduced from 2D spectra (HSQC and HMBC).

**NMR:** see Figure S2

1. Ac-Aib_1_-Pro_2_-Aib_3_-Ala_4_-Aib_5_-Ala_6_-Gln_7_-Aib_8_-Val_9_-Aib_10_-Gly_11_-Leu_12_-Aib_13_-Pro_14_-Val_15_-Aib_16_-Aib_17_-Gln_18_-Gln_19_-Phaol (Sept. B)

**ABCD 25** was dissolved in methanol saturated with NH_3_. Further NH_3_ is bubbled through the solution every 7 days. After 30 days, the solvent is evaporated. For HPLC see figure S3.

**ESI**: 669.4032 [M + 3H]^3+^, 1003.6043 [M + 2H]^2+^, 2006.18600 [M + H]^+^ (see Figure S4)

Synthesis of analogues of Septocylindrin B

1. Cbz-Glu(OMe)_19_-Phaol_20_-N6-Boc

The product is synthesized using the general method for coupling reactions described above. The product is purified *via* MPLC: DCM:MeOH 100:0🡪99:1.

**Cbz-Phaol-N6-Boc**

(0.3 g, 0.65 mmol), Cbz-Glu(OMe)-OH (0.25 g, 1.3 eq, 0.84 mmol); EDC.HCl (0.16 g, 1.3 eq, 0.84 mmol); HOAt (0.11 g, 1.3 eq, 0.84 mmol); NMM (0.15 ml, 2 eq, 1.3 mmol).

**Yield**: 77 % (0.3 g)

**Melting point**: 76-78 °C

**IR** (cm^-1^)**:** 3305, 2971, 2929, 1736, 1692, 1675, 1653, 1536.

**^1^H NMR** (600 MHz, CDCl_3_, ppm): δ = 7.37-7.19 (m, 10H, Ph), 7.01 (s, 1H, NH_20_), 5.54 (s, 1H, NH_19_), 5.14-5.08 (m, 2H, C*H*_2_ Cbz), 4.20 (br s, 2H, αH_19,20_), 3.64 (s, 3H, Me_19_), 3.60 (m, 1H, αCHC*H*_2_N), 3.18 (br s, 0.5H, NC*H_2_*CH_2_), 3.09-3.08 (m, 1H, NC*H_2_*CH_2_), 2.99-2.92 (m, 2.5H, βH_20_ + NC*H_2_*CH_2_ + αCHC*H*_2_N), 2.37-2.33 (m, 2H, γH_19_), 2.09 + 1.88 2*(br s, 1H, βH_19_), 1.44 (s, 9H, C(C*H_3_*)_3_), 1.38-1.19 (m, 8H, NCH_2_C*H_2_*C*H_2_*C*H_2_*C*H_2_*), 0.87 (t, *J* = 7 Hz, 3H, Me_20_).

**^13^C NMR** (100 MHz, CDCl_3_, ppm): δ = 173.3 (δCO_19_), 170.9 (CO_19_), 157.4 (CO Boc), 156.0 (CO Cbz), 137.5 (C Phe), 136.3 (C Cbz), 129.2, 128.5, 128.1, 128.0, 126.6 (CH Ph), 80.1 (*C*(CH_3_)_3_), 67.0 (*C*H_2_Cbz), 54.2 (αC_19_), 51.7 (Me_19_), 51.4 (αC_20_), 49.1 (αCH*C*H_2_N), 47.8 (NH*C*H_2_CH_2_), 38.9 (βC_20_), 31.3 + 28.2 + 26.3 + 22.5 (NCH_2_*C*H_2_*C*H_2_*C*H_2_*C*H_2_), 30.0 (γC_19_), 28.3 (C(*C*H_3_)_3_), 28.2 (βC_19_), 14.0 (Me_20_).

**ESI:** 635.0 [M + Na]^+^, 1245.8 [2M + Na]^+^

1. Cbz-Glu(OMe)_18_-Glu(OMe)_19_-Phaol_20_-N6-Boc

The product is synthesized using the general method for coupling reactions described above. The product is purified *via* MPLC: DCM:MeOH 100:0🡪98:2.

**Cbz-Glu(OMe)-Phaol-N6-Boc 27** (0.25 g, 0.41 mmol), Cbz-Glu(OMe)-OH (0.16 g, 1.3 eq, 0.53 mmol); EDC.HCl (0.1 g, 1.3 eq, 0.53 mmol); HOAt (0.07 g, 1.3 eq, 0.53 mmol); NMM (0.1 ml, 2 eq, 0.8 mmol).

**Yield**: 84 % (0.26 g)

**Melting point**: 90-93 °C

**IR** (cm^-1^)**:** 3286, 3065, 2980, 2930, 1736, 1685, 1640, 1537.

**^1^H NMR** (600 MHz, CDCl_3_, ppm): δ = 7.36-7.19 (m, 10H, Ph), 7.03 (s, 2H, NH_19,20_), 5.74 (s, 1H, NH_18_), 5.15-5.09 (m, 2H, C*H*_2_ Cbz), 4.41 (br s, 1H, αH_19_), 4.24 (br s, 2H, αH_18,20_), 3.68 + 3.64 2*(s, 3H, Me_18,19_), 3.53-3.52 (m, 1H, αCHC*H*_2_N), 3.28 (m, 1H, NC*H_2_*CH_2_), 3.09 (m, 0.5H, NC*H_2_*CH_2_), 3.03 (m, 1.5H, NC*H_2_*CH_2_ + αCHC*H*_2_N), 2.95-2.93 + 2.73 2*(m, 1H, βH_20_), 2.51-2.41 (m, 2H, γH_18_), 2.29 (m, 2H, γH_19_), 2.13 + 1.99-1.97 2*(m, 1H, βH_18_), 2.06 + 1.90 2*(m, 1H, βH_19_), 1.44 (s, 9H, C(C*H_3_*)_3_), 1.40-1.21 (m, 8H, NCH_2_C*H_2_*C*H_2_*C*H_2_*C*H_2_*), 0.88 (t, *J* = 7 Hz, 3H, Me_20_).

**^13^C NMR** (150 MHz, CDCl_3_, ppm): δ = 173.8 + 173.6 (δCO_18,19_), 170.9 + 170.4 (CO_18,19_), 157.1 (CO Boc), 156.3 (CO Cbz), 137.7 (C Phe), 136.2 (C Cbz), 129.2 + 128.5 + 128.4 + 128.2 + 128.1 + 126.5 (CH Ph), 79.9 (*C*(CH_3_)_3_), 67.1 (*C*H_2_ Cbz), 54.4 (αC_18_), 52.3 (αC_19_), 51.9 + 51.7 (Me_18,19_), 51.2 (αC_20_), 49.2 (αCH*C*H_2_N), 47.7 (NH*C*H_2_CH_2_), 38.7 (βC_20_), 31.4 + 28.3 + 26.3 + 22.5 (NCH_2_*C*H_2_*C*H_2_*C*H_2_*C*H_2_), 30.2 (γC_18_), 29.9 (γC_19_), 28.3 (C(*C*H_3_)_3_), 27.9 (βC_18_), 27.7 (βC_19_), 14.0 (Me_20_).

**ESI:** 778.2 [M + Na]^+^, 1531.9 [2M + Na]^+^

1. Cbz-Pro_14_-Val_15_-Aib_16_-Aib_17_-Glu(OMe)_18_-Glu(OMe)_19_-Phaol_20_-N3-Boc (Cbz-C^3^D^2^-Phaol-N6-Boc)

The product is synthesized using the general method for coupling reactions described above. **Cbz-C^3^-O*t*Bu 19** is *t*butyl deprotected with the deprotection method using TFA described above. The product is purified *via* MPLC: DCM:MeOH 100:0🡪96:4.

**Cbz-Glu(OMe)-Glu(OMe)-Phaol-N6-Boc 28** (0.15 g, 0.2 mmol), **Cbz-C^3^-OH** (0.13 g, 1.2 eq, 0.25 mmol); EDC.HCl (0.05 g, 1.2 eq, 0.25 mmol); HOAt (0.034 g, 1.2 eq, 0.25 mmol); NMM (0.5 ml, 2 eq, 0.4 mmol).

**Yield**: 79 % (0.19 g)

**Melting point:** 49-52 °C

**IR** (cm^-1^)**:** 3294, 2980, 2931, 1738, 1649, 1530.

**^1^H NMR** (600 MHz, CDCl_3_, ppm): δ = 7.76 (s, 1H, NH_18_), 7.61 (d, *J* = 8 Hz, 1H, NH_19_), 7.53 (s, 1H, NH_16_), 7.34-7.19 (m, 10H, Ph), 7.11 (s, 2H, NH_17_), 6.94 (d, *J* = 8 Hz, 1H, NH_20_), 6.66 (s, 1H, NH_15_), 5.22-5.10 (m, 2H, C*H*_2_ Cbz), 4.39 (br s, 1H, αH_20_), 4.24-4.23 (m, 2H, αH_14,19_), 4.04-4.03 (m, 1H, αH_18_), 3.80-3.79 (m, 1H, αH_15_), 3.60-3.59 (m, 9H, δH_14_ + αCHC*H*_2_N + Me_18,19_), 3.38 + 3.26 2*(br s, 0.5H, NC*H_2_*CH_2_), 3.11-3.08 (m, 2H, αCHC*H*_2_N + NC*H_2_*CH_2_), 2.85 + 2.65 2*(m, 1H, βH_20_), 2.53-2.49 (m, 2H, γH_18,19_), 2.28 (m, 4H, βH_14_ + γH_18,19_), 2.18-2.12 (m, 2H, βH_18,19_), 2.02-1.96 (m, 5H, βH_15,18,19_ + γH_14_), 1.54-1.24 (m, 29H, βH_16,17_ + NCH_2_C*H_2_*C*H_2_*C*H_2_*C*H_2_ +* C(C*H_3_*)_3_), 0.84-0.83 (m, 6H, Me_20_ + γH_15_), 0.79 (d, *J* = 7 Hz, 3H, γH_15_).

**^13^C NMR** (150 MHz, CDCl_3_, ppm): δ = 176.6 + 175.2 (CO_Aib_), 173.5 + 173.3 + 173.1 (CO_14_ + δCO_18,19_), 172.2 (CO_18_), 171.6 (CO_15_), 171.1 (CO_19_), 156.3 (CO Cbz), 155.5 (CO Boc), 138.8 (C Phe), 136.1 (C Cbz), 129.4 + 128.7 + 128.5 + 128.4 + 128.1 + 128.0 + 125.9 (CH Ph), 79.2 (*C*(CH_3_)_3_), 67.8 (*C*H_2_ Cbz), 62.0 (αC_14_), 61.0 (αC_15_), 55.3 (αC_18_), 53.3 (αC_19_), 51.5 + 51.3 (Me_18,19_), 50.6 (αCH*C*H_2_N), 49.9 (αC_20_), 48.0 (NH*C*H_2_CH_2_), 47.3 (δC_14_), 39.0 (βC_20_), 31.6 + 28.4 + 27.9 + 22.6 (NCH_2_*C*H_2_*C*H_2_*C*H_2_*C*H_2_), 31.0 + 30.8 (γC_18,19_), 28.4 (C(*C*H_3_)_3_), 27.4 + 26.8 + 23.1 + 22.9 (βC_16,17_), 26.5 (βC_19_), 26.0 (βC_18_), 18.8 + 18.2 (γH_15_), 14.0 (Me_20_).

**ESI:** 1144.3 [M + Na]^+^

1. Cbz-Aib_5_-Ala_6_-Glu(OMe)_7_-Aib_8_-Val_9_-Aib_10_-Gly_11_-Leu_12_-Aib_13_-Pro_14_-Val_15_-Aib_16_-Aib_17_-Glu(OMe)_18_-Glu(OMe)_19_-Phaol_20_-N6-Boc (Cbz-BC^3^D^2^-Phaol-N6-Boc)

The product is synthesized using the general method for coupling reactions described above. **Cbz-B-O*t*Bu 16** is *t*butyl deprotected with the deprotection method using TFA described above. The product is purified *via* MPLC: DCM:MeOH 100:0🡪92:8.

**Cbz-C^3^D^2^** **-Phaol-N6-Boc 29** (0.16 g, 0.14 mmol), **Cbz-B-OH** (0.165 g, 1.3 eq, 0.19 mmol); EDC.HCl (0.035 g, 1.3 eq, 0.19 mmol); HOAt (0.025 g, 1.3 eq, 0.19 mmol); NMM (0.03 ml, 2 eq, 0.18 mmol).

**Yield**: 27 % (0.07 g)

**^1^H NMR** (600 MHz, CDCl_3_, ppm): δ = 7.35-7.14 (m, Ph), 5.12 (s, 2H, C*H*_2_ Cbz), 1.43 (m, C(C*H_3_*)_3_).

**^13^C NMR** (150 MHz, CDCl_3_, ppm): δ = 129.5 + 128.6 + 128.2 + 128.1 127.4 + 126.0 (CH Ph), 67.0 (*C*H_2_ Cbz), 31.6 + 29.4 + 26.5 + 22.7 (NCH_2_*C*H_2_*C*H_2_*C*H_2_*C*H_2_), 28.5 (C(*C*H_3_)_3_).

**ESI:** 953.6 [M + 2Na]^2+^, 1882.9 [M + Na]^+^

**NMR:** see figure S5

1. Ac-Aib_1_-Pro_2_-Aib_3_-Ala_4_-Aib_5_-Ala_6_-Glu(OMe)_7_-Aib_8_-Val_9_-Aib_10_-Gly_11_-Leu_12_-Aib_13_-Pro_14_-Val_15_-Aib_16_-Aib_17_-Glu(OMe)_18_-Glu(OMe)_19_-Phaol_20_-N6-Boc (ABC^3^D^2^-N6-Boc)

The product is synthesized using the general method for coupling reactions described above. The mixture solvent used for the coupling reaction is DCM:DMSO 1:1. The product is purified *via* HPLC.

**Cbz-BC^3^D^2^-Phaol-N6-Boc 30** (0.06 g, 0.03 mmol), **A-OH** 9 (0.02 g, 1.3 eq, 0.04 mmol); PyBOP (0.022 g, 1.3 eq, 0.04 mmol); NMM (0.007 ml, 2 eq, 0.06 mmol).

**Yield**: 33 % (0.023 g)

**ESI:** 1118.668 [M + 2Na]^2+^, 2192.336 [M + H]^+^, 2214.319 [M + Na]^+^

1. Ac-Aib_1_-Pro_2_-Aib_3_-Ala_4_-Aib_5_-Ala_6_-Glu(OMe)_7_-Aib_8_-Val_9_-Aib_10_-Gly_11_-Leu_12_-Aib_13_-Pro_14_-Val_15_-Aib_16_-Aib_17_-Glu(OMe)_18_-Glu(OMe)_19_-Phaol_20_-N6 (ABC^3^D^2^-Phaol-N6)

**ABC^3^D^2^-Phaol-N6-Boc 31** (0.022 g, 0.01 mmol) is dissolved in dry dichloromethane. 5 eq. of BiCl_3_ (0.0016 g, 5 eq, 0.05 mmol) were added. The reaction is stirred overnight at reflux temperature. Water is added and the pH is made neutral. The mixture is extracted with dichloromethane. The product is purified *via* HPLC (see Figure S6).

**Yield**: 7 % (0.0015 g)

**ESI:** 1046.619 [M + 2H]^2+^, 2092.249 [M + H]^+^, 2214.319 [M + Na]^+^

1. Cbz-Glu(OMe)_19_-Phe_20_-ethanolamine-TBDMS

The product is synthesized using the general method for coupling reactions described above. The product is purified *via* MPLC: heptane:EtOAc 60:40🡪40:60.

**Cbz-Phe-ethanolamine-TBDMS** (0.3 g, 0.67 mmol), Cbz-Glu(OMe)-OH (0.26 g, 1.3 eq, 0.87 mmol); EDC.HCl (0.17 g, 1.3 eq, 0.87 mmol); HOAt (0.12 g, 1.3 eq, 0.87 mmol); NMM (0.15 ml, 2 eq, 1.3 mmol).

**Yield**: 88 % (0.35 g)

**Melting point**: 129-131 °C

**IR** (cm^-1^)**:** 3293, 3063, 2951, 2929, 2857, 1735, 1696, 1643, 1537.

**^1^H NMR** (600 MHz, CDCl_3_, ppm): δ = 7.33-7.18 (m, 10H, Ph), 7.01 (s, 1H, NH_20_), 6.10 (s, 1H, NH_ethanolamine_), 5.78 (s, 1H, NH_19_), 5.13-5.06 (m, 2H, CH_2_ Cbz), 4.61-4.60 (m, 1H, αH_20_), 4.27-4.26 (m, 1H, αH_19_), 3.66 (s, 3H, Me_19_), 3.58-.355 + 3.50-3.49 2*(m, 1H, CH_2_O), 3.31-3.26 (m, 2H, NCH_2_), 3.05 (d, *J* = 7 Hz, 2H, βH_20_), 2.43-2.34 (m, 2H, γH_19_), 2.11-2.05 + 1.95-1.92 2*(m, 1H, βH_19_), 0.87 (s, 9H, C(C*H_3_*)_3_), 0.02 (s, 6H, 2* Me_TBDMS_).

**^13^C NMR** (100 MHz, CDCl_3_, ppm): δ = 173.8 (δC_19_), 170.9 (CO_19_), 170.2 (CO_20_), 156.3 (CO Cbz), 136.5 + 136.1 (C Phe + C Cbz), 129.2 + 128.6 + 128.5 + 128.2 + 128.1 + 127.0 (CH Ph), 67.1 (CH_2_ Cbz), 61.4 (CH_2_O), 54.8 (αC_20_), 54.4 (αC_19_), 51.9 (Me_19_), 41.7 (NCH_2_), 38.7 (βC_20_), 30.1 (γC_19_), 27.8 (βC_19_), 25.9 (C(*C*H_3_)_3_), 18.2 (*C*(CH_3_)_3_), -5.4 (Me).

**ESI:** 623.2 [M + Na]^+^, 1222.0 [2M + Na]^+^

1. Cbz-Glu(OMe)_18_-Glu(OMe)_19_- Phe_20_-ethanolamine-TBDMS

The product is synthesized using the general method for coupling reactions described above. The product is purified *via* MPLC: DCM:MeOH 100:0🡪97:3.

**Cbz-Glu(OMe)-Phe-ethanolamine-TBDMS 33** (0.77 g, 1.3 mmol), Cbz-Glu(OMe)-OH (0.49 g, 1.3 eq, 1.67 mmol); EDC.HCl (0.32 g, 1.3 eq, 1.67 mmol); HOAt (0.23 g, 1.3 eq, 1.67 mmol); NMM (0.3 ml, 2 eq, 2.6 mmol).

**Yield**: 80 % (0.76 g)

**Melting point**: 142-145 °C

**IR** (cm^-1^)**:** 3278, 3066, 3031, 2952, 2930, 2857, 1737, 1687, 1687, 1664, 1635, 1534.

**^1^H NMR** (600 MHz, CDCl_3_, ppm): δ = 8.11 (NH_19_), 7.88 + 7.01 (NH_20_ + NH_ethanolamine_), 7.35-7.17 (m, 10H, Ph), 6.49 (s, 1H, NH_18_), 5.13-5.01 (m, 2H, CH_2_ Cbz), 4.91-4.89 (m, 1H, αH_20_), 4.79 (m, 1H, αH_19_), 4.56 (m, 1H, αH_18_), 3.63 + 3.60 2*(s, 3H, Me_18,19_), 3.56-3.44 (m, 2H, CH_2_O), 3.36-3.31 (m, 2H, NCH_2_), 3.20-3.14 (m, 2H, βH_20_ + NCH_2_), 3.05-3.02 (m, 1H, βH_20_), 2.46-2.30 (m, 4H, γH_18,19_), 2.11-1.98 (m, 4H, βH_18,19_), 0.86 (s, 9H, C(C*H_3_*)_3_), 0.02 (s, 6H, 2* Me_TBDMS_).

**^13^C NMR** (100 MHz, CDCl_3_, ppm): δ = 173.6 + 173.5 (δC_18,19_), 171.7 (CO_18_), 170.8 + 170.7 (CO_19,20_), 156.5 (CO Cbz), 136.9 (C Phe), 136.2 (C Cbz), 129.3 + 128.5 + 128.3 + 128.1 + 128.0 + 126.7 (CH Ph), 67.0 (CH_2_ Cbz), 61.5 (CH_2_O), 54.5 + 54.4 (αC_18,20_), 52.5 (αC_19_), 51.7 (Me_18,19_), 41.8 (NCH_2_), 39.0 (βC_20_), 30.1 + 30.0 (γC_18,19_), 28.4 + 28.1 (βC_18,19_), 25.9 (C(*C*H_3_)_3_), 18.2 (*C*(CH_3_)_3_), -5.4 (Me).

**ESI:** 766.1 [M + Na]^+^, 1508.1 [2M + Na]^+^

1. Cbz-Pro_14_-Val_15_-Aib_16_-Aib_17_-Glu(OMe)_18_-Glu(OMe)_19_- Phe_20_-ethanolamine-TBDMS (Cbz-CD-Phe-ethanolamine-TBDMS)

The product is synthesized using the general method for coupling reactions described above. **Cbz-C-O*t*Bu 19** is *t*butyl deprotected with the deprotection method using TFA described above. The product is purified *via* MPLC: DCM:MeOH 100:0🡪96:4.

**Cbz-Glu(OMe)-Glu(OMe)-Phe-ethanolamine-TBDMS 34** (0.15 g, 0.2 mmol), **Cbz-C-OH** (0.1 g, 1 eq, 0.2 mmol); EDC.HCl (0.04 g, 1 eq, 0.2 mmol); HOAt (0.03 g, 1 eq, 0.2 mmol); NMM (0.04 ml, 2 eq, 0.4 mmol).

**Yield**: 55 % (0.12 g)

**Melting point**: 57-61 °C

**IR** (cm^-1^)**:** 3329, 2980, 2930, 1739, 1650, 1531.

**^1^H NMR** (600 MHz, CDCl_3_, ppm): δ = 7.85 (d, *J* = 5 Hz, NH_18_), 7.75 (d, *J* = 6 Hz, NH_19_), 7.51 (NH_Aib_), 7.37-7.13 (m, 13H, Ph + NH_20_ + NH_ethanolamine_ + NH_Aib_), 6.55 (br s, 1H, NH_15_), 5.23-5.12 (m, 2H, CH_2_ Cbz), 4.73 (m, 1H, αH_20_), 4.24 (t, *J* = 7 Hz, 1H, αH_14_), 4.13 (m, 1H, αH_19_), 4.01 (m, 1H, αH_18_), 3.81 (s, 1H, αH_15_), 3.71 (m, 2H, CH_2_O), 3.64-3.62 (m, 8H, δH_14_ + Me_18,19_), 3.56-3.54 + 2.86-2.82 2*(m, 1H, βH_20_), 3.39-3.37 (m, 2H, NCH_2_), 2.59-2.56 + 2.53-2.47 2*(m, 1H, γH_18,19_), 2.33-2.20 (m, 7H, γH_18,19_ + βH_14,18,19_), 2.03-1.92 (m, 4H, βH_14,15_ + γH_14_), 1.48-1.42 (m, 12H, βH_16,17_), 0.88 (s, 9H, C(C*H_3_*)_3_), 0.85 + 0.81 2*(d, *J* = 7 Hz, 3H, γH_15_), 0.06 (s, 6H, 2* Me_TBDMS_).

**^13^C NMR** (150 MHz, CDCl_3_, ppm): δ = 176.8 (CO_17_), 175.3 (CO_16_), 173.0 + 172.9 (δC_18,19_), 173.5 + 171.7 + 171.5 (CO_14,15,18,19,20_), 156.4 (CO Cbz), 138.4 (C Phe), 136.0 (C Cbz), 129.1 + 128.7 + 128.6 + 128.1 + 128.1 + 126.2 (CH Ph), 67.9 (CH_2_ Cbz), 62.1 (αC_14_), 61.4 (CH_2_O), 61.1 (αC_15_), 55.7 (αC_18_), 54.8 (αC_20_), 54.6 (αC_19_), 51.5 + 51.4 (Me_18,19_), 47.4 (δC_14_), 41.9 (NCH_2_), 37.6 (βC_20_), 31.0 + 30.5 (γC_18,19_), 30.0 (βC_14_), 29.0 (βC_15_), 27.4 + 26.8 + 22.9 (βC_16,17_), 26.0 (C(*C*H_3_)_3_), 25.8 (βC_18,19_), 24.8 (γC_14_), 18.8 + 18.3 (γH_15_), 18.3 (*C*(CH_3_)_3_), -5.3 (Me).

**ESI:** 766.1 [M + Na]^+^, 1508.1 [2M + Na]^+^

1. Ac-Aib_1_-Pro_2_-Aib_3_-Ala_4_-Aib_5_-Ala_6_-Glu(OMe)_7_-Aib_8_-Val_9_-Aib_10_-Gly_11_-Leu_12_-Aib_13_-OtBu (AB-O*t*Bu)

The product is synthesized using the coupling method described above. The product is purified *via* MPLC: dichloromethane: MeOH 100:0 🡪94:6.

**Cbz-B-O*t*Bu 16** (0.44 g, 0.47 mmol); **A-OH** 9 (0.23 g, 1 eq, 0.47 mmol); EDC.HCl (0.9 g, 1 eq, 0.47 mmol); HOAt (0.06 g, 1 eq, 0.47 mmol); NMM (0.1 ml, 2 eq, 0.47 mmol).

**Yield:** 67% (1.34 g)

**Melting point:** 135-140 °C

**IR** (cm^-1^)**:** 3657, 3293, 2981, 1886, 2362, 2342, 1736, 1648, 1529.

**^1^H NMR** (600 MHz, CDCl_3_, ppm): δ = 8.31 + 7.80 + 7.67 + 7.54 + 7.28 + 4*(s, 1H, NH_Aib_), 7.91 (m, 3H, NH_11,12,Aib_), 7.85 (s, 1H, NH_7_), 7.63 + 7.48 2*(s, 1H, NH_4,6_), 4.39-4.35 (αH_12_), 4.20 (t, *J* = 8 Hz, 1H, αH_2_), 4.07-4.03 (m, 3H, αH_4,6,11_ + δH_2_), 3.97-3.96 (m, 1H, αH_4,6_), 3.92-3.88 (m, 1H, αH_7_), 3.64-3.62 (m, 3H, αH_11_ + Me_7_), 3.39-3.37 (m, 1H, δH_2_), 2.73-2.68 (m, 1H, γH_7_), 2.45-2.35 (m, 3H, βH_2_ + γH_7_), 2.32-2.27 (m, 1H, βH_7_), 2.23-2.19 (m, 2H, βH_7,9_), 2.11 (s, 3H, Me_Ac_), 2.09 (m, 1H, γH_2_), 1.97-1.89 (m, 3H, βH_12_ + γH_2,12_), 1.65-1.42 (m, 64H, βH_Aib,4,6,12_ + C(C*H*_3_)_3_), 1.11 + 1.00 2*(d, *J* = 6 Hz, 3H, γH_9_), 0.93-0.89 (m, 6H, δH_12_).

**^13^C NMR** (150 MHz, CDCl_3_, ppm): δ = 177-170.6 (CO), 172.7 (CO_7_), 171.1 (CO_Ac_), 80.1 (*C*(CH_3_)_3_), 64.5 (αC_2_), 63.8 (αC_9_), 56.7-56.1 (αC_7,Aib_), 52.8 + 52.3 (αC_4,6_), 52.7 (αC_12_), 51.6 (Me_7_), 49.0 (δC_2_), 44.3 (αC_11_), 40.5 (βC_12_), 30.8 (γC_7_), 29.2 (βC_9_), 28.9 (βC_2_), 27.8 (C(*C*H_3_)_3_), 27.1 + 27.0 + 26.9 + 26.5 + 26.1 + 24.3 + 23.9 + 23.4 + 22.8 + 22.8 + 22.7 + 22.6 (βC_Aib_), 26.3 (γC_2_), 25.6 (βC_7_),24.2 (γH_12_), 23.2 + 21.1 (δC_12_), 22.6 (Me_Ac_), 20.3 + 19.3 (γC_9_), 16.8 + 16.6 (βC_4,6_).

**ESI:** 1667.9 [M + Na]^+^

1. Ac-Aib_1_-Pro_2_-Aib_3_-Ala_4_-Aib_5_-Ala_6_-Glu(OMe)_7_-Aib_8_-Val_9_-Aib_10_-Gly_11_-Leu_12_-Aib_13_-Pro_14_-Val_15_-Aib_16_-Aib_17_-Glu(OMe)_18_-Glu(OMe)_19_- Phe_20_-ethanolamine (ABCD-Phe-ethanolamine)

**ABCD-Phe-ethanolamine-TBDMS** is synthesized using the coupling method described above. **AB-O*t*Bu 36** is *t*butyl deprotected with the deprotection method using ZnBr_2_ described above.

**Cbz-CD-Phe-ethanolamine-TBDMS 35** (0.17 g, 0.15 mmol); **AB-OH** (0.22 g, 1.2 eq, 0.18 mmol); PyBOP (0.1 g, 1.2 eq, 0.18 mmol); NMM (0.04 ml, 2 eq, 0.3 mmol).

**ABCD-Phe-ethanolamine-TBDMS** (impure, 0.1 g, 0.05 mmol) is dissolved in dry THF and cooled to -78 °C. TBAF (0.07 ml, 1.5 eq, 0.07 mmol) is added and the reaction is stirred for 1 hour at room temperature. The mixture is made neutral with KHSO_4_ solution and extracted with dichloromethane. The product is purified *via* HPLC (see Figure S7).

**Yield:** 43% (0.034 g; over coupling and deprotection step)

**ESI:** 1063.532 [M + 2Na]^2+^, 2104.088 [M + Na]^+^

1. Cbz-Glu(OMe)_19_-Phaol_20_-N5O-Boc_2_

The product is synthesized using the coupling method described above. The product is purified *via* MPLC: dichloromethane: MeOH 100:0 🡪98:2.

**Cbz-Phaol-N5O-Boc_2_** (0.72 g, 1.3 mmol); Cbz-Glu(OMe)-OH (0.49 g, 1.3 eq, 1.7 mmol); EDC.HCl (0.32 g, 1.3 eq, 1.7 mmol); HOAt (0.22 g, 1.3 eq, 1.7 mmol); NMM (0.3 ml, 2 eq, 2.5 mmol).

**Yield**: 99 % (0.89 g)

**Melting point**: oil

**IR** (cm^-1^)**:** 3317, 3291, 3064, 3032, 2980, 1735, 1688, 1675, 1651, 1561, 1530.

**^1^H NMR** (300 MHz, CDCl_3_, ppm): δ = 7.35-7.18 (m, 10H, Ph), 6.93 (s, 1H, NH_20_), 5.50 (br s, 1H, NH_19_), 5.15-5.05 (m, 2H, C*H*_2_Cbz), 4.17 + 4.03-3.99 (m, 3H, αH_19,20_+ CH_2_O), 3.75 (s, 1H, αCHC*H*_2_N + NC*H*_2_CH_2_), 3.66-3.64 (m, 4H, αCHC*H*_2_N + NC*H*_2_CH_2_ + Me_19_), 3.13-3.08 (m, 1H, αCHC*H*_2_N + NC*H*_2_CH_2_), 2.98-2.81 (m, 2H, αCHC*H*_2_N + NC*H*_2_CH_2_ + βH_20_), 2.68 (m, 1H, βH_20_), 2.42-2.32 (m, 2H, γH_19_), 2.05 + 1.88-1.81 2*(m, 1H, βH_19_), 1.58 + 1.43 + 1.26 3*(br s, 2H, NCH_2_C*H_2_*C*H_2_*C*H_2_*), 1.49 + 1.42 2*(s, 9H, C(C*H*_3_)_3_).

**^13^C NMR** (75 MHz, CDCl_3_, ppm): δ = 173.5 (δCO_19_), 170.9 (CO_19_), 157.6 + 156.3 + 153.6 (CO Boc + CO Cbz), 137.4 (*C* Phe), 136.3 (*C* Cbz), 129.2 + 128.5 + 128.1 + 128.1 + 126.6 (CH Ph), 81.9 + 80.2 (*C*(CH_3_)_3_), 67.0 + 66.8 (*C*H_2_ Cbz +*C*H_2_O), 54.1 (αC_19_), 51.7 + 51.5 (αC_20_ + Me_19_), 49.0 (αCH*C*H_2_N), 47.5 (NH*C*H_2_CH_2_), 38.8 (βC_20_), 30.0 + 28.0 + 27.8 + 23.0 (*C*H_2_*C*H_2_*C*H_2_CH_2_O + γC_19_ + βC_19_), 28.3 + 27.8 (C(*C*H_3_)_3_).

**ESI:** 736.9 [M + Na]^+^

1. Cbz-Glu(OMe)_18_-Glu(OMe)_19_-Phaol_20_-N5O-Boc_2_

The product is synthesized using the coupling method described above. The product is purified *via* MPLC: heptane:EtOAc 70:30 🡪30:70.

**Cbz-Glu(OMe)-Phaol-N5O-Boc_2_ 38** (0.89 g, 1.2 mmol); Cbz-Glu(OMe)-OH (0.4 g, 1.3 eq, 1.6 mmol); EDC.HCl (0.31 g, 1.3 eq, 1.6 mmol); HOAt (0.22 g, 1.3 eq, 1.6 mmol); NMM (0.3 ml, 2 eq, 2.5 mmol).

**Yield**: 69 % (0.74 g)

**Melting point**: oil

**IR** (cm^-1^)**:** 3297, 2961, 1734, 1686, 1670, 1633, 1536, 1515.

**^1^H NMR** (400 MHz, CDCl_3_, ppm): δ = 7.47-7.20 (m, 10H, Ph), 7.00 (m, 2H, NH_19,20_), 5.73 (br s, 1H, NH_18_), 5.13-5.09 (m, 2H, C*H*_2_ Cbz), 4.40 + 4.24 (m, 3H, αH_18,19,20_), 4.05-4.02 (m, 2H, CH_2_O), 3.70-6.65 (m, 6H, Me_18,19_), 3.56-3.53 + 3.26 + 3.12 + 3.05 (m, 4H, αCHC*H*_2_N + NC*H*_2_CH_2_), 2.92 + 2.75 2*(m, 1H, βH_20_), 2.49-2.40 + 2.29 2*(m, 2H, γH_18,19_), 2.13-1.94 (m, 4H, βH_19_), 1.61 + 1.60 (C*H_2_*CH_2_O), 1.50-1.38 (m, 20 H, C(C*H*_3_)_3_+ NCH_2_CH_2_C*H_2_*), 1.29 (br s, 2H, NCH_2_C*H_2_*).

**^13^C NMR** (150 MHz, CDCl_3_, ppm): δ = 173.2 (δCO_18,19_), 171.0 + 170.4 (CO_18,19_), 157.0 (CO *N-*Boc), 156.3 (CO Cbz), 153.6 (CO O-Boc), 137.7 (C Phe), 136.2 (*C* Cbz), 129.2 + 128.5 + 128.5 + 128.2 + 128.0 + 126.5 (CH Ph), 81.8 + 80.2 (*C*(CH_3_)_3_), 67.1 + 66.8 (*C*H_2_ Cbz +*C*H_2_O), 54.5 + 52.6 (αC_18,19_), 51.9 + 51.8 (Me_18,19_), 51.1 (αC_20_), 49.3 (αCH*C*H_2_N), 47.4 (NH*C*H_2_CH_2_), 38.7 (βC_20_), 30.2 + 30.0 (γC_18,19_), 28.4+ 27.8 (C(*C*H_3_)_3_), 28.3 + 28.0 (*C*H_2_*C*H_2_CH_2_O), 27.6 + 26.9 (βC_19_), 23.0 (NHCH_2_*C*H_2_).

**ESI:** 880.3 [M + Na]^+^

1. Ac-Aib_1_-Pro_2_-Aib_3_-Ala_4_-Aib_5_-Ala_6_-Glu(OMe)_7_-Aib_8_-Val_9_-Aib_10_-Gly_11_-Leu_12_-Aib_13_-Pro_14_-Val_15_-Aib_16_-Aib_17_-Glu(OMe)_18_-Glu(OMe)_19_-Phaol_20_- N5O-Boc_2_ (ABCD-N5O-Boc_2_)

The product is synthesized using the coupling method described above. It is purified *via* HPLC. **ABC-O*t*Bu 23** is *t*butyl deprotected with the deprotection method using ZnBr_2_ described above.

**Cbz-D-Phaol-N5O-Boc_2_ 39** (0.1 g, 1.2 eq, 0.11 mmol); **ABC-OH** (0.15 g, 1 eq, 0.09 mmol); PyBOP (0.054 g, 1.1 eq, 0.10 mmol); NMM (0.02 ml, 2 eq, 0.19 mmol).

**Yield:** 40% (0.086 g)

**ESI:** 1158.637 [M + H+Na]^2+^, 1169.634 [M + 2Na]^2+^, 1177.618 [M + Na+K]^2+^, 1185.600 [M + 2K]^2+^, 2294.290 [M + H]^+^_,_ 2316.265 [M + Na]^+^, 2332.252 [M + K]^+^

1. Ac-Aib_1_-Pro_2_-Aib_3_-Ala_4_-Aib_5_-Ala_6_-Glu(OMe)_7_-Aib_8_-Val_9_-Aib_10_-Gly_11_-Leu_12_-Aib_13_-Pro_14_-Val_15_-Aib_16_-Aib_17_-Glu(OMe)_18_-Glu(OMe)_19_-Phaol_20_- N5O (ABC^3^D^2^-N5O)

**ABCD-Phaol-N5O-Boc_2_ 40** (0.079 g, 0.035 mmol), is dissolved in dry dichloromethane. 5 eq. of BiCl_3_ (0.055 g, 5 eq, 0.17 mmol) are added. The reaction is stirred overnight at reflux temperature. Water is added and the mixture is made neutral. The mixture is extracted with dichloromethane. The product is purified *via* HPLC (see Figure S8).

**Yield**: 12 % (0.0086 g)

**ESI:** 1047.588 [M + 2H]^2+^, 1058.582 [M + H + Na]^2+^, 2094.170 [M + H]^+^

1. Cbz-Glu(OMe)_19_-Alaol_20_-Boc_2_

The product is synthesized using the coupling method described above. The product is purified *via* MPLC: heptane:EtOAc 70:30 🡪50:50.

**Cbz-Alaol-Boc_2_** (0.6 g, 1.3 mmol); Cbz-Glu(OMe)-OH (0.5 g, 1.3 eq, 1.7 mmol); EDC.HCl (0.3 g, 1.3 eq, 1.7 mmol); HOAt (0.24 g, 1.3 eq, 1.7 mmol); NMM (0.3 ml, 2 eq, 2.7 mmol).

**Yield:** 85 % (0.68 g)

**Melting point**: oil

**IR** (cm^-1^)**:** 3308, 2980, 2361, 1738, 1694, 1657, 1530.

**^1^H NMR** (400 MHz, CDCl_3_, ppm): δ = 7.33-7.28 (m, 5H, Ph), 6.96 (s, 1H, NH_20_), 5.59 (d, *J* = 6 Hz, 1H, NH_19_), 5.12-5.10 (m, 2H, C*H*_2_ Cbz), 4.17-4.15 (m, 3H, C*H_2_*O + αH_19_), 3.08 (br s, 1H, αH_20_),3.66 (s, 3H, Me_19_), 3.60-3.57 + 3.10-3.06 2*(m, 1H, αCHC*H*_2_NH), 3.50-3.48 + 3.40-3.38 2*(m, 1H, NHC*H*_2_CH_2_), 2.41-2.36 (m, 2H, γH_19_), 2.14-2.08 + 1.93-1.90 2*(m, 1H, βH_19_), 1.50 + 1.47 (2*s, 18H, C(C*H*_3_)_3_), 1.15-1.13 (m, 3H, βH_20_).

**^13^C NMR** (100 MHz, CDCl_3_, ppm): δ = 173.4 (δCO_19_), 170.7 (CO_19_), 156.9 (CO *N-*Boc), 156.0 (CO Cbz), 153.3 (CO O-Boc), 136.3 (C Cbz), 128.5 + 128.1 + 128.0 (CH Cbz), 82.4 + 80.8 (*C*(CH_3_)_3_), 66.9 (CH_2_ Cbz), 64.6 (CH_2_O), 54.1 (αC_19_), 52.3 (αCH*C*H_2_NH), 51.7 (Me_19_), 46.9 (NH*C*H_2_CH_2_), 46.1 (αC_20_), 29.9 (γC_19_), 28.5 (βC_19_), 28.3 + 27.7 (C(*C*H_3_)_3_), 18.2 (βC_20_).

**ESI:** 619.3 [M + Na]^+^, 1213.6 [2M + Na]^+^

1. Cbz-Glu(OMe)_18_-Glu(OMe)_19_-Alaol_20_-Boc_2_

The product is synthesized using the coupling method described above. The product is purified *via* MPLC: heptane:EtOAc 60:40 🡪25:75.

**Cbz-Glu(OMe)-Alaol-Boc_2_ 42** (0.7 g, 1.1 mmol); Cbz-Glu(OMe)-OH (0.4 g, 1.3 eq, 1.5 mmol); EDC.HCl (0.28 g, 1.3 eq, 1.5 mmol); HOAt (0.2 g, 1.3 eq, 1.5 mmol); NMM (0.2 ml, 2 eq, 2.3 mmol).

**Yield:** 46 % (0.39 g)

**Melting point**: 147-150 °C

**IR** (cm^-1^)**:** 3284, 2980, 2889, 1737, 1691, 1672, 1632, 1560, 1528.

**^1^H NMR** (400 MHz, CDCl_3_, ppm): δ = 7.37-7.28 (m, 5H, Ph), 7.04 + 6.96 2*(br s, 1H, NH_19,20_), 5.71 (s, 1H, NH_18_), 5.13 (s, 2H, C*H*_2_ Cbz), 4.41-4.40 (m, 1H, αH_19_), 4.25-4.12 (m, 4H, C*H_2_*O + αH_18,20_), 3.68 + 3.66 2*(s, 3H, Me_18,19_), 3.51-3.41 (m, 3H, αCHC*H*_2_NH + NHC*H*_2_CH_2_), 3.27-3.13 (m, 1H, αCHC*H*_2_NH), 2.49-2.36 (m, 4H, γH_18,19_), 2.18-2.14 + 2.00-1.95 2*(m, 2H, βH_18,19_), 1.49 + 1.46 (2*s, 18H, C(C*H*_3_)_3_), 1.14-1.12 (m, 3H, βH_20_).

**^13^C NMR** (100 MHz, CDCl_3_, ppm): δ = 173.7 + 173.5 (δCO_18,19_), 171.1 + 170.2 (CO_18,19_), 156.3 (CO *N-*Boc), 156.3 (CO Cbz), 153.3 (CO O-Boc), 136.2 (C Cbz), 128.5 + 128.2 + 128.0 (CH Cbz), 82.3 + 80.7 (*C*(CH_3_)_3_), 67.1 (CH_2_ Cbz), 64.6 (CH_2_O), 54.5 (αC_19_), 52.6 (αC_18_), 52.2 (αCH*C*H_2_NH), 51.9 + 51.7 (Me_18,19_), 46.8 (NH*C*H_2_CH_2_), 45.9 (αC_20_), 30.2 + 30.0 (γC_19_), 28.3 + 27.7 (C(*C*H_3_)_3_), 27.9 + 27.7 (βC_18,19_), 18.2 (βC_20_).

**ESI:** 762.3 [M + Na]^+^, 1499.5 [2M + Na]^+^

1. Ac-Aib_1_-Pro_2_-Aib_3_-Ala_4_-Aib_5_-Ala_6_-Glu(OMe)_7_-Aib_8_-Val_9_-Aib_10_-Gly_11_-Leu_12_-Aib_13_-Pro_14_-Val_15_-Aib_16_-Aib_17_-Glu(OMe)_18_-Glu(OMe)_19_-Alaol_20_-Boc_2_ (ABCD-Alaol-Boc_2_)

The product is synthesized using the coupling method described above. It is purified *via* HPLC. **ABC-O*t*Bu 23** is *t*butyl deprotected with the deprotection method using TFA described above.

**Cbz-D-Alaol-Boc_2_ 43** (0.084 g, 1.2 eq, 0.11 mmol); **ABC^3^-OH** (0.15 g, 1 eq, 0.09 mmol); PyBOP (0.054 g, 1.1 eq, 0.10 mmol); NMM (0.02 ml, 2 eq, 0.19 mmol).

**Yield:** 50% (0.104 g)

**ESI:** 1110.607 [M + 2Na]^2+^, 2176.223 [M + H]^+^_,_ 2198.201 [M + Na]^+^

1. Ac-Aib_1_-Pro_2_-Aib_3_-Ala_4_-Aib_5_-Ala_6_-Glu(OMe)_7_-Aib_8_-Val_9_-Aib_10_-Gly_11_-Leu_12_-Aib_13_-Pro_14_-Val_15_-Aib_16_-Aib_17_-Glu(OMe)_18_-Glu(OMe)_19_-Alaol_20_ (ABCD-Alaol)

**ABCD-Alaol-Boc_2_ 44** (0.096 g, 0.044 mmol), is dissolved in dry dichloromethane. 5 eq. of BiCl_3_ (0.07 g, 5 eq, 0.22 mmol) were added. The reaction is stirred overnight at reflux temperature. Water is added and the mixture is made neutral. The mixture is extracted with dichloromethane. The product is purified *via* HPLC (see Figure S9).

**Yield**: 29 % (0.0025 g)

**ESI:** 988.559 [M + 2H]^2+^, 1976.135 [M + H]^+^

1. Ac-Aib_1_-Pro_2_-Aib_3_-Ala_4_-Aib_5_-Ala_6_-Glu(OMe)_7_-Aib_8_-Val_9_-Aib_10_-Gly_11_-Leu_12_-Aib_13_-Pro_14_-Val_15_-Aib_16_-Aib_17_-Glu(OMe)_18_-Glu(OMe)_19_-Alaol_20_-Boc (ABCD-Alaol-Boc)

This product is isolated as a side product of the Boc-deprotection of **ABC^3^D^2^-Alaol-Boc_2_ 44**.

**Yield**: 11 % (0.0049 g)

**ESI:** 1060.564 [M + 2Na]^2+^, 2076.158 [M + H]^+^, 2098.144 [M + Na]^+^, 2114.117 [M + K]^+^

Leakage assay

To a solution of 7.8 mM PC (5 mL) in distilled DCM, 368 µL of a 45.38 mM solution of Ch in distilled DCM is added. The lipid micelles (in a PC:Ch 7:3 ratio) are dried by a flow of nitrogen gas and put in a desiccator for three hours. Meanwhile a 50 mM solution of CF in a Hepes buffer is prepared by dissolving 113 mg of CF (used without purification) in 4 mL water. A 1 N NaOH solution is added until a pH of 7.4 is reached, then 1 mL of a 30 mM Hepes buffer solution pH 7.4 and 1 mL of water is added. The final pH should be situated between 7.4 and 7.5. The lipid micelles are hydrated by 6 mL of the 50 mM CF solution and stirred with a glass stirrer. The lipid suspension is left standing in the dark at room temperature for 15 hours. The following day, the suspension is sonicated at 0 °C for 45 minutes. The SUVs and free CF are separated by exclusion chromatography on a Sephadex G-75 column (diameter 2 cm, height 60 cm), using a 5 mM Hepes solution, pH 7.4 as eluent. The dilution factor for the lipid solution is calculated. The phospholipid concentration is kept constant throughout the experiments ([PC+Ch] = 60 µM). Increasing [peptide]/[lipid] ratios (R^-1^) are obtained by adding aliquots of MeOH solutions of peptides, keeping the final MeOH concentration below 5% by volume. After rapid and vigorous stirring, the time-course of fluorescence change corresponding to CF escape was recorded at 520 nM (6 nm band pass), with λexc 488 nm (3 nm band pass). The percentage of released CF at time t was determined by:

%CF = (F_t_ - F_0_)/(F_T_ – F_0_) x 100%

With: F_0_ = intensity of fluorescence of the liposomes in absence of peptide.

F_t_ = intensity of fluorescence at time t in the presence of peptide.

F_T_ = intensity of fluorescence after liposome destruction by adding 50 µL 10% Triton X-100 in water.
